# Glucocerebrosidase reduces the spread of protein aggregation in a *Drosophila melanogaster* model of neurodegeneration by regulating proteins trafficked by extracellular vesicles

**DOI:** 10.1371/journal.pgen.1008859

**Published:** 2021-02-04

**Authors:** Kathryn A. Jewett, Ruth E. Thomas, Chi Q. Phan, Bernice Lin, Gillian Milstein, Selina Yu, Lisa F. Bettcher, Fausto Carnevale Neto, Danijel Djukovic, Daniel Raftery, Leo J. Pallanck, Marie Y. Davis

**Affiliations:** 1 Department of Genome Sciences, University of Washington, Seattle, Washington, United States of America; 2 Department of Biology, Juniata College, Huntingdon, Pennsylvania, United States of America; 3 VA Puget Sound Healthcare System, Seattle, Washington, United States of America; 4 Northwest Metabolomics Research Center, Department of Anesthesiology and Pain Medicine, University of Washington, Seattle, Washington, United States of America; 5 Department of Neurology, University of Washington, Seattle, Washington, United States of America; NHGRI, UNITED STATES

## Abstract

Abnormal protein aggregation within neurons is a key pathologic feature of Parkinson’s disease (PD). The spread of brain protein aggregates is associated with clinical disease progression, but how this occurs remains unclear. Mutations in *glucosidase*, *beta acid 1* (*GBA*), which encodes glucocerebrosidase (GCase), are the most penetrant common genetic risk factor for PD and dementia with Lewy bodies and associate with faster disease progression. To explore how *GBA* mutations influence pathogenesis, we previously created a *Drosophila* model of *GBA* deficiency (*Gba1b*) that manifests neurodegeneration and accelerated protein aggregation. Proteomic analysis of *Gba1b* mutants revealed dysregulation of proteins involved in extracellular vesicle (EV) biology, and we found altered protein composition of EVs from *Gba1b* mutants. Accordingly, we hypothesized that *GBA* may influence pathogenic protein aggregate spread via EVs. We found that accumulation of ubiquitinated proteins and Ref(2)P, *Drosophila* homologue of mammalian p62, were reduced in muscle and brain tissue of *Gba1b* flies by ectopic expression of wildtype GCase in muscle. Neuronal GCase expression also rescued protein aggregation both cell-autonomously in brain and non-cell-autonomously in muscle. Muscle-specific *GBA* expression reduced the elevated levels of EV-intrinsic proteins and Ref(2)P found in EVs from *Gba1b* flies. Perturbing EV biogenesis through neutral sphingomyelinase (nSMase), an enzyme important for EV release and ceramide metabolism, enhanced protein aggregation when knocked down in muscle, but did not modify *Gba1b* mutant protein aggregation when knocked down in neurons. Lipidomic analysis of nSMase knockdown on ceramide and glucosylceramide levels suggested that *Gba1b* mutant protein aggregation may depend on relative depletion of specific ceramide species often enriched in EVs. Finally, we identified ectopically expressed GCase within isolated EVs. Together, our findings suggest that GCase deficiency promotes accelerated protein aggregate spread between cells and tissues via dysregulated EVs, and EV-mediated trafficking of GCase may partially account for the reduction in aggregate spread.

## Author summary

Parkinson’s disease (PD) is a common neurodegenerative disease characterized by abnormal clumps of proteins (aggregates) within the brain and other tissues which can lead to cellular dysfunction and death. Mutations in the gene *GBA*, which encodes glucocerebrosidase (GCase), are the strongest genetic risk factor for PD, and are associated with faster disease progression. GCase-deficient mutant flies display features suggestive of PD including increased protein aggregation in brain and muscle. We found that restoring GCase protein in the muscle of mutant flies reduced protein aggregation in muscle and the brain, suggesting a mechanism involving interaction between tissues. Previous work indicated that *GBA* influences extracellular vesicles (EVs)–small membrane-bound structures released by cells to communicate and/or transport cargo from cell to cell. Here, we found increased aggregated proteins within EVs of mutant flies, which was reduced by restoring GCase in muscle. In addition, we found GCase within the EVs, possibly explaining how GCase in one tissue such as muscle could reduce protein aggregation in a distant tissue like the brain. Our findings suggest that GCase influences proteins within EVs, affecting the spread of protein aggregation. This may be important to understanding PD progression and could uncover new targets to slow neurodegeneration.

## Introduction

Parkinson’s disease (PD) is the most common neurodegenerative movement disorder, affecting 1–2% of people over 65 years of age [1]. PD is characterized by cardinal motor and non-motor symptoms, including rigidity, slowness of voluntary movements, and cognitive decline [2–4]. Intraneuronal Lewy bodies containing ubiquitinated proteins and α-synuclein are a hallmark pathologic finding in PD. The stereotypic spread of Lewy bodies in PD from the rostral brain stem to the midbrain and eventually throughout the neocortex suggests a prion-like mechanism mediating propagation of protein aggregates from neuron to neuron [5]. This temporo-spatial spread of Lewy bodies correlates with clinical progression of PD and has been replicated in several animal models [6–8]. Although much work has focused on identifying genes involved in PD, and how perturbation of these genes lead to PD pathogenesis, the mechanisms underlying PD are not yet completely understood.

Mutations in the gene *glucosidase*, *beta acid 1* (*GBA*), encoding the lysosomal ceramide metabolism enzyme glucocerebrosidase (GCase), are the strongest genetic risk factor for PD and dementia with Lewy bodies, increasing risk of developing PD by approximately 5-fold in *GBA* mutation carriers compared to non-carriers [9–11]. *GBA* carriers with PD are otherwise clinically similar to idiopathic PD patients, with indistinguishable response to dopaminergic medications, slightly younger age of onset by about 4 years, and higher incidence of cognitive decline [12,13]. While some studies suggest that *GBA* carriers with PD may have a heavier burden of Lewy bodies than in non-carriers with PD, the neuropathologic features are similar [14]. Importantly, mutations in the gene *GBA* are common, having been found in 4–5% of all idiopathic PD patients [13,15]. Recent longitudinal clinical studies have revealed that in addition to increased risk, *GBA* carriers with PD have faster progression of both motor and cognitive symptoms compared to idiopathic PD patients [16–18].

Several models have been developed and characterized using *Drosophila melanogaster* to examine how *GBA* influences PD pathogenesis [19–22]. Our previously characterized *Drosophila* model of *GBA* deficiency (*Gba1b*) manifests several phenotypes reminiscent of key features of PD, including neurodegeneration, locomotor deficits, cognitive deficits, and accelerated protein aggregation in multiple tissues including the nervous system and muscle [19]. Proteomic analysis of our *Gba1b* mutant flies revealed that proteins involved in extracellular vesicle (EV) biology were dysregulated, and EVs isolated from *Gba1b Drosophila* hemolymph revealed increased levels of aggregate-prone and EV-intrinsic proteins, indicating that *GBA* deficiency alters the protein composition of EVs [23]. EVs are a heterogeneous group of membrane-bound vesicles secreted by cells that can have multiple functions, including intercellular communication through protein, lipid, and nucleic acid cargo and discard of cell components outside of the cell [24,25]. EVs can originate from the endosomal system as a result of fusion of the late endosome to the plasma membrane, releasing intraluminal vesicles as EVs into the extracellular matrix (exosomes), or from direct outward budding of the plasma membrane (microvesicles) [24,26,27]. EVs have been implicated in the propagation of misfolded proteins between cells in multiple neurodegenerative diseases, including PD [28–33]. α-synuclein has been found within EVs isolated from tissues of PD and dementia with Lewy bodies patients [28,34], and *in vitro* studies have suggested that α-synuclein within EVs may be more pathogenic than free extracellular α-synuclein [35]. Accordingly, we hypothesized that tissue-specific expression of wildtype (WT) GCase might reduce the protein aggregates that accumulate in *Gba1b* mutants.

Using our *GBA*-deficient *Drosophila* model [18,23], we examined whether GCase could be mediating propagation of protein aggregates from cell-to-cell via EVs. In this study, we found that tissue-specific expression of WT GCase in *Gba1b* mutants corrected the alterations in protein composition of *GBA*-deficient EVs, as well as protein aggregation in local and distant tissues. Perturbing an EV biogenesis pathway in *Gba1b* mutants by knocking down *neutral sphingomyelinase* (*nSMase*), encoding an enzyme important for EV release as well as ceramide metabolism, resulted in tissue-specific cell-autonomous effects on protein aggregation and changes in EV protein composition but did not rescue protein aggregation in distant tissues. Lipidomic analysis of the effect of *nSMase* knockdown on ceramide (Cer) and glucosylceramide (GlcCer) levels suggested that *Gba1b* mutant protein aggregation may be dependent on relative depletion of specific Cer species that are known to be enriched in EVs. Finally, we observed ectopically expressed GCase in EVs, suggesting that trafficking of GCase within EVs may contribute to the observed non-cell-autonomous rescue. Our findings suggest that mutations in *GBA* result in alterations in the lipid composition of cells and the protein composition of EVs that promote enhanced cell-to-cell transmission of pathogenic protein aggregates. Moreover, our findings indicate that GCase can be packaged into EVs and trafficked between cells to reduce protein aggregation throughout an organism. These findings suggest a possible mechanism underlying the clinical finding that *GBA* mutation carriers not only have increased risk of developing PD, but also faster progression of disease.

## Results

### Protein aggregation in *Gba1b* mutants can be rescued cell-autonomously and non-cell-autonomously by tissue-specific *dGba1b* expression

**Fig 1:**
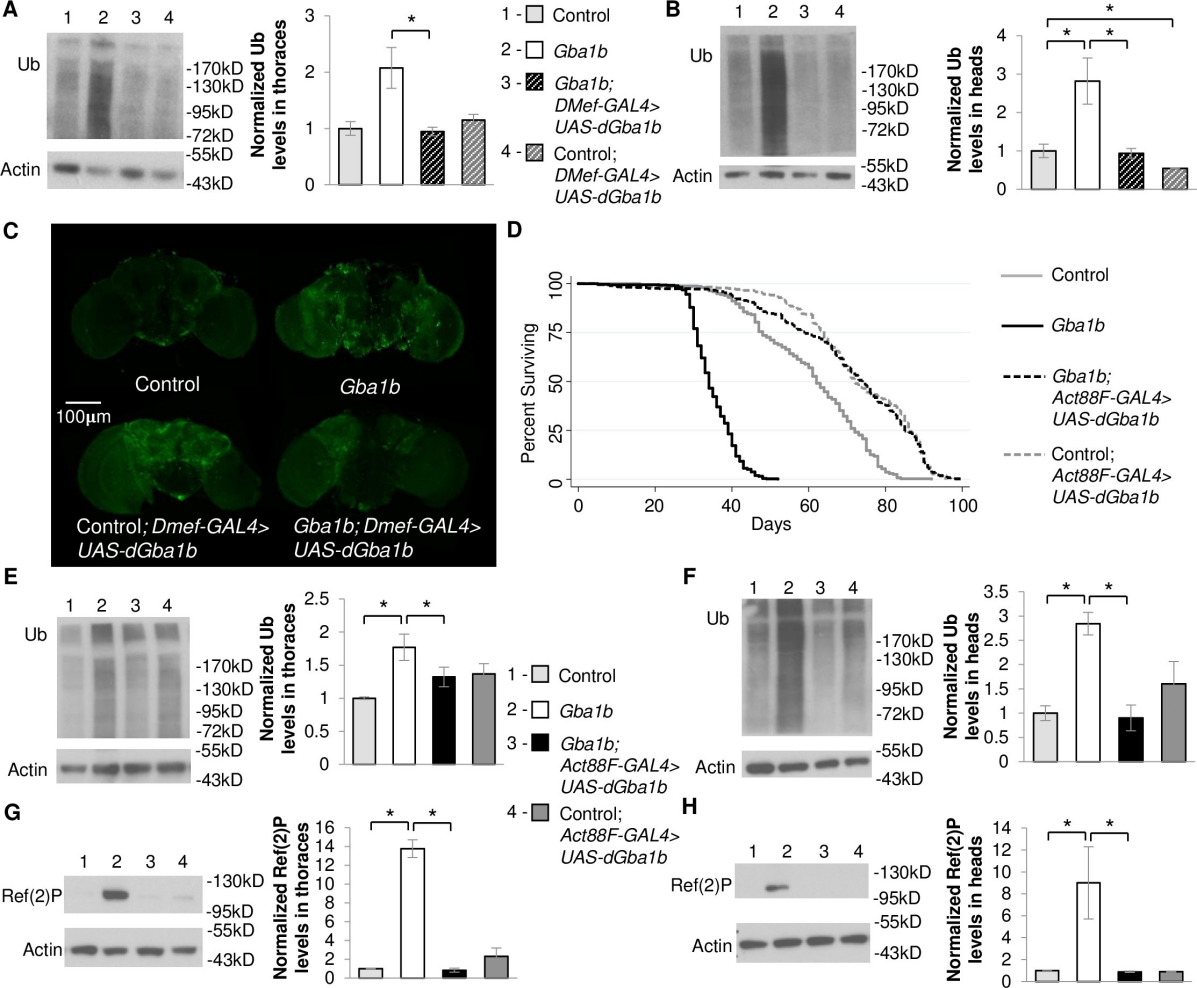
Muscle expression of *dGba1b* rescues protein aggregation and lifespan in *Gba1b* mutants. (A-C) Using the muscle-specific driver, *DMef-GAL4*, wildtype (WT) *Drosophila Gba1b* (*dGba1b)* was expressed in *Gba1b* mutants and WT revertant controls. (A,B) Homogenates were prepared from fly thoraces and heads using 1% Triton X-100 lysis buffer. Western blot analysis was performed on the Triton X-100 insoluble proteins using antibodies to ubiquitin (Ub) and Actin. Representative images and quantification of ubiquitin (Ub) in (A) thoraces (one-way ANOVA: F(3,7) = 6.972, p = 0.016) and (B) heads (F(3,7) = 8.822, p = 0.009) are shown. Results are normalized to Actin loading control and control flies. (C) Representative anti-Ub immunofluorescent staining of whole brains from control, *Gba1b* mutant, and control and *Gba1b* mutants expressing *dGba1b* using the *DMef-GAL4* driver. (D-H) Using the indirect flight muscle specific driver, *Act88F-GAL4*, WT *dGba1b* was expressed in *Gba1b* mutants and wildtype revertant controls. (D) Kaplan-Meier survival curves of control, *Gba1b* mutants, and *Gba1b* mutants and controls expressing WT *dGba1b* using the *Act88F-GAL4* driver. (E-H) Homogenates were prepared from fly thoraces and heads using 1% Triton X-100 lysis buffer. Western blot analysis was performed on the Triton X-100 insoluble proteins using antibodies to ubiquitin (Ub) and Actin, and on the soluble fractions using antibodies to Ref(2)P and Actin. Representative images and quantification of ubiquitin in (E) thoraces (F(3,8) = 4.657, p = 0.036) and (F) heads (F(3,8) = 9.538, p = 0.005) and Ref(2)P in (G) thoraces (F(3,8) = 88.77, p < 0.001) and (H) heads (F(3,8) = 5.977, p = 0.019) of controls and *Gba1b* mutants with and without muscle expression of WT *dGba1b* are shown. Results are normalized to Actin in controls. At least 3 independent experiments were performed. Error bars represent SEM. *p < 0.05 by Student t-test.

Our prior work revealed accelerated aggregate accumulation and dysregulation of EV-related proteins in *Gba1b* mutant flies suggesting that GCase deficiency may influence cell-to-cell spread of protein aggregates. To explore the hypothesis that mutations in *GBA* promote the spread of protein aggregates from peripheral tissues to the brain, we expressed WT *Drosophila Gba1b* (*dGba1b)* in non-neural tissues of *Gba1b* mutants and examined accumulation of ubiquitinated protein and Ref(2)P, the *Drosophila* homologue of mammalian p62, in the brain. Using the *DMef-GAL4* driver to drive expression of WT *dGba1b* in muscles throughout the fly, insoluble ubiquitinated protein aggregate accumulation was reduced in both the thoraces and heads of *Gba1b* mutant flies (Fig 1A and 1B). This non-cell-autonomous rescue of ubiquitinated protein aggregates was dramatically apparent in the whole brains of *Gba1b* mutants (Fig 1C). However, because *DMef-GAL4* is also expressed in muscles located in the fly head that control the proboscis and may not exclusively drive expression in only muscle cells, we repeated this experiment using the *Act88F-GAL4* driver. *Act88F* expression is specifically in the thoracic indirect flight muscles and is not found in the head [36]. Expression of WT *dGba1b* using the *Act88F-GAL4* driver in *Gba1b* mutants significantly reduced accumulation of insoluble ubiquitinated proteins in the thoraces and heads (Fig 1E and 1F) and rescued their shortened lifespan (Fig 1D and S1 Table).

Ref(2)P, the *Drosophila* ortholog of mammalian SQSTM1/p62, is important for selectively targeting ubiquitinated proteins for lysosomal degradation. While Ref(2)P is often used as a marker of autophagic flux, we have previously found it to accumulate in *Gba1b* mutants with no evidence of significantly impaired autophagy [23]. However, Ref(2)P is also required for aggregate formation under normal physiological conditions [37], and it has been shown to be released concomitantly with α-synuclein in EVs [34]; therefore we used its accumulation as a second readout of protein aggregation. We anticipated that Ref(2)P accumulation in *Gba1b* mutants could also be non-cell-autonomously rescued. We indeed found that Ref(2)P accumulation was significantly decreased in both the thoraces and heads of *Gba1b* mutants expressing WT *dGba1b* in flight muscle (Fig 1G and 1H).

**Fig 2:**
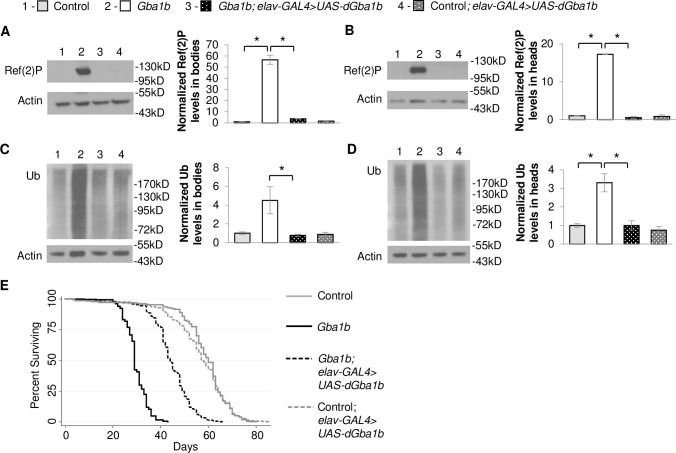
Neuronal expression of *dGba1b* rescues protein aggregation in *Gba1b* mutants. (A-D) Using the neuronal driver *elav-GAL4*, wildtype *dGba1b* was expressed in *Gba1b* mutants and wildtype revertant controls. Homogenates were prepared from fly heads and bodies using 1% Triton X-100 lysis buffer. Western blot analysis was performed on the Triton X-100 soluble fractions using antibodies to Ref(2)P and Actin and on the insoluble proteins using antibodies to ubiquitin (Ub) and Actin. Representative images and quantification of Ref(2)P in (A) bodies (one-way ANOVA: F(3,7) = 153.581, p < 0.001) and (B) heads (F(3,6) = 542.043, p < 0.001) and Ub in (C) bodies (F(3,8) = 6.099, p = 0.018) and (D) heads (F(3,8) = 16.878, p < 0.001) of control and *Gba1b* mutants with and without neuronal expression of *dGba1b* are shown. Results are normalized to Actin and control. At least 3 independent experiments were performed. Error bars represent SEM. *p < 0.05 by Student t-test. (E) Kaplan-Meier survival curves of control, *Gba1b* mutants, and *Gba1b* mutants and controls expressing WT *dGba1b* using the *elav-GAL4* driver.

To determine whether there might be a directionality or tissue-specificity to the non-cell-autonomous rescue of protein aggregation, we expressed WT *dGba1b* in the nervous system and assessed for protein aggregates in the body. Neuronal expression of WT *dGba1b* using the *elav*-*GAL4* driver decreased both Ref(2)P accumulation and insoluble ubiquitinated proteins in both the heads and bodies of *Gba1b* mutants (Fig 2A–2D). In addition, neuronal expression of WT *dGba1b* partially rescued the shortened lifespan of *Gba1b* mutant flies (Fig 2E and S2 Table). Our findings indicate that *GBA* has both cell-autonomous and non-cell-autonomous roles in preventing protein aggregation. The non-cell-autonomous role of *GBA* raises the possibility that GCase may normally function to reduce the spread of protein aggregates from one cell and tissue to another.

### Non-cell-autonomous rescue of protein aggregation in *Gba1b* mutants is mediated by extracellular vesicles

**Fig 3:**
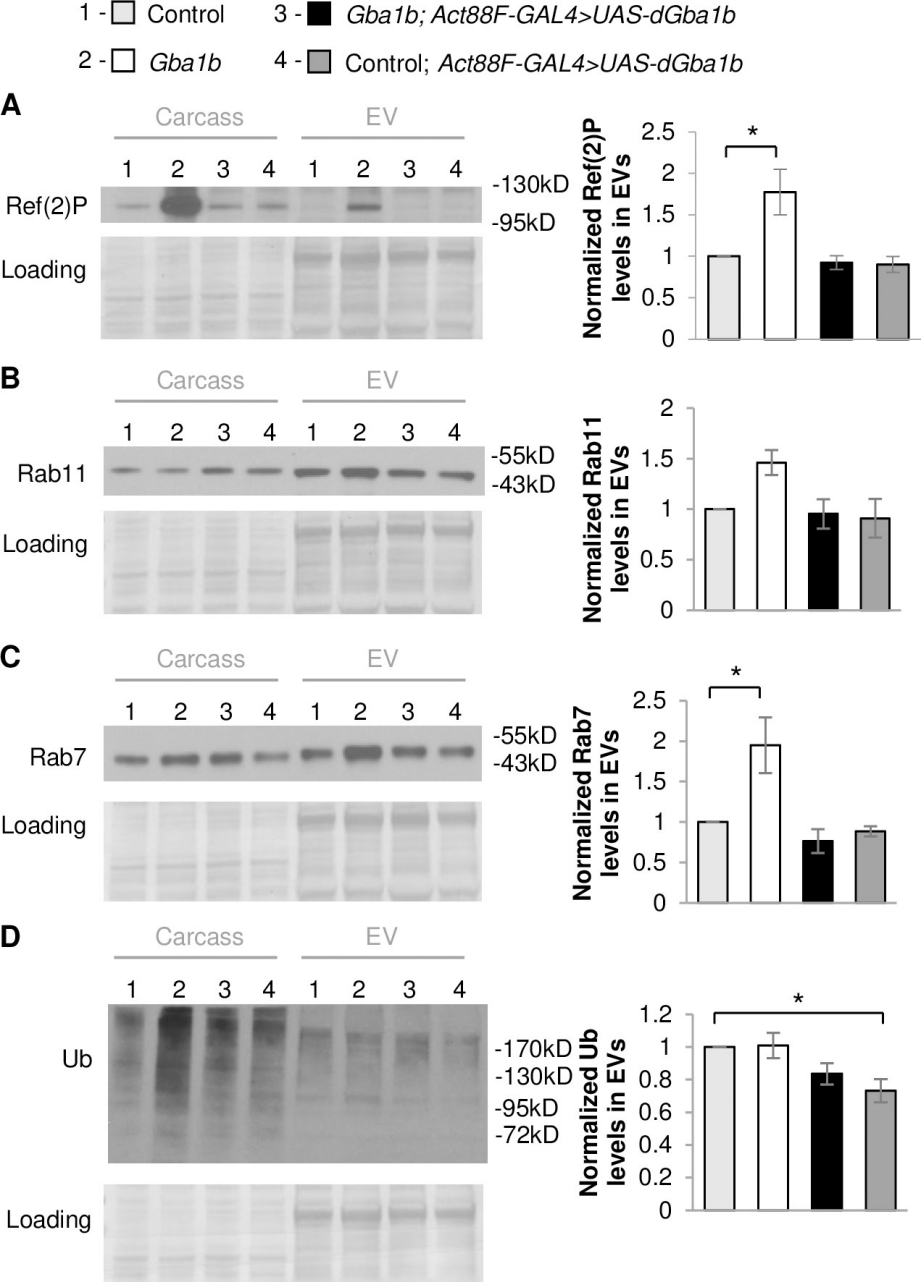
Muscle expression of *dGba1b* rescues alterations in EV-intrinsic proteins and Ref(2)P observed in EVs. (A-D) Fly carcass homogenates and isolated extracellular vesicles (EVs) from *Gba1b* mutants and wildtype revertant control flies with and without wildtype *dGba1b* expressed in indirect flight muscle using the *Act88F-GAL4* driver. Western blot analysis was performed using antibodies to (A) Ref(2)P (One-way ANOVA: F(3,8) = 7.610, p = 0.010), (B) Rab11 (F(3,8) = 3.584, p = 0.066), (C) Rab7 (F(3,8) = 8.133, p = 0.008), and (D) ubiquitin (Ub) (F(3,8) = 4.718, p = 0.035). Representative images and quantification normalized to controls are shown. Ponceau S is shown to verify equal loading. At least 3 independent experiments were performed. Error bars represent SEM. *p < 0.05 by Student t-test.

There are multiple mechanisms that could mediate non-cell-autonomous interactions, including exocytosis of cytoplasmic components into the extracellular matrix, direct cell-to-cell contacts, and release of cytoplasmic components via EVs. Given that our prior proteomic analysis of *Gba1b* mutants found evidence of dysregulation of EVs [23], we hypothesized that GCase influences the trafficking of aggregate-prone proteins in EVs. We isolated EVs from hemolymph and confirmed that Ref(2)P was increased in EVs from *Gba1b* mutants compared to controls (Fig 3A), as we had seen previously [23]. We also found that EV-intrinsic proteins Rab11 and Rab7 were elevated, although the increase in Rab11 did not reach statistical significance (Fig 3B and 3C). We tested the possibility that muscle-specific expression of WT *dGba1b* would reduce the increased levels of Ref(2)P and EV-intrinsic proteins from *Gba1b* mutants. Indeed, we observed a reduction in Ref(2)P and Rab7 levels in EVs isolated from *Gba1b* mutants expressing WT *dGba1b* in flight muscle using the *Act88F-Gal4* driver (Fig 3A and 3C). However, while ubiquitinated proteins were increased in *Gba1b* mutant whole flies, they were not significantly altered in EVs isolated from *Gba1b* mutants (Fig 3D). These results indicate that expression of GCase in the muscles of *Gba1b* mutants restores normal EV content and EVs may promote the spread of protein aggregates between tissues.

### Tissue-specific Cer depletion is necessary for the spread of protein aggregation in *Gba1b* mutants

**Fig 4:**
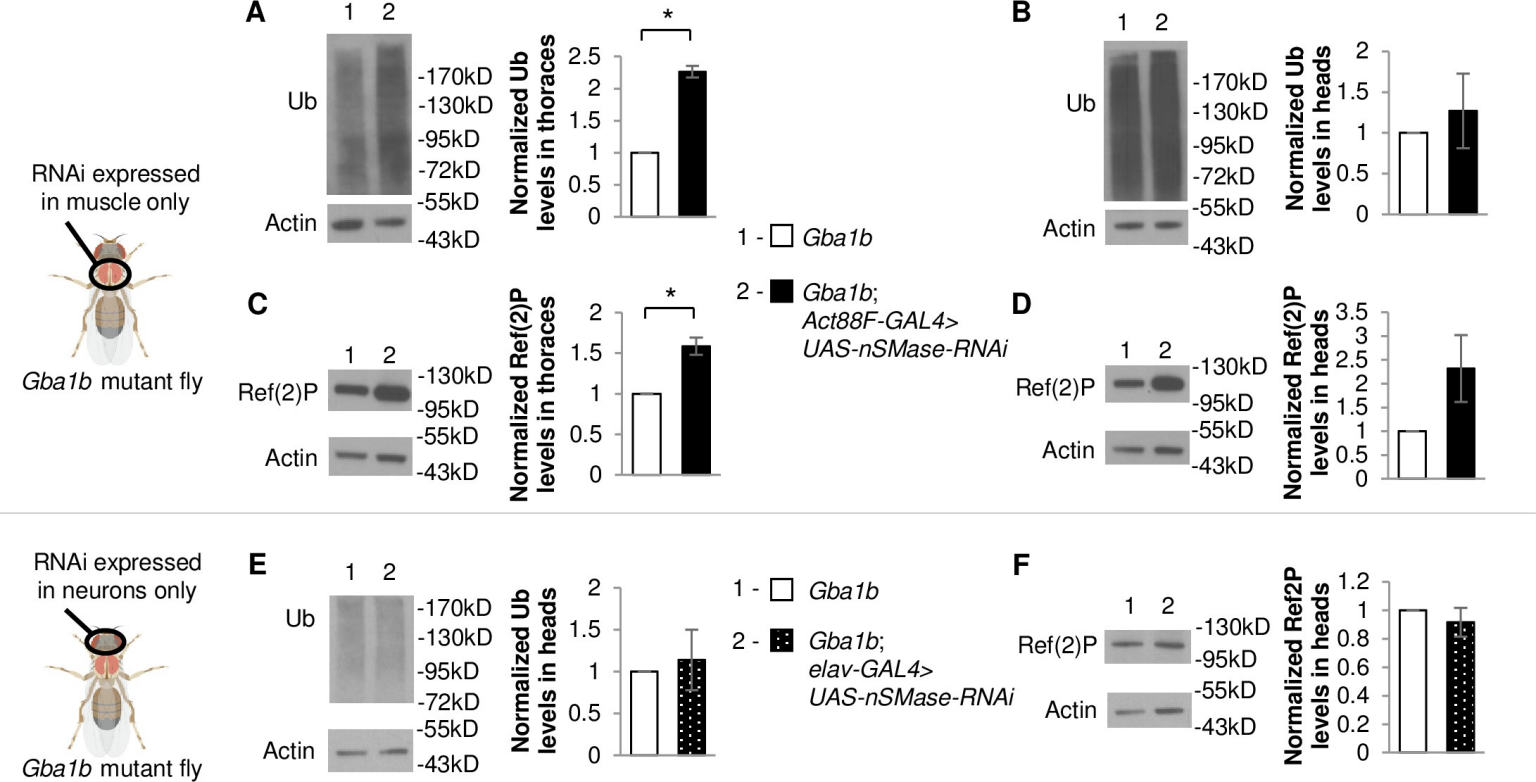
Tissue-specific knockdown of nSMase in *Gba1b* mutants differentially alters ubiquitinated proteins and Ref(2)P. (A-F) *Neutral sphingomyelinase (nSMase)-*RNAi was expressed using tissue-specific drivers in *Gba1b* mutants. Homogenates were prepared from fly heads and thoraces using 1% Triton X-100 lysis buffer. Western blot analysis was performed on the Triton X-100 insoluble proteins using antibodies to ubiquitin (Ub) and Actin, and on the soluble fractions using antibodies to Ref(2)P and Actin. (A-D) Representative images and quantification of ubiquitin in (A) thoraces and (B) heads and Ref(2)P in (C) thoraces and (D) heads of flies with and without nSMase knockdown in flight muscle using the *Act88F-GAL4* driver. (E,F) Representative images and quantification of (E) ubiquitin and (F) Ref(2)P in the heads of flies with and without nSMase knockdown in neurons using the *elav-GAL4* driver. Results are normalized to *Gba1b* mutants without RNAi expression. At least 3 independent experiments were performed. Error bars represent SEM. *p < 0.05 by Student t-test.

To examine whether EVs may be mediating the observed non-cell-autonomous rescue, we examined factors influencing the metabolism of EVs. We hypothesized that disruption of EV biogenesis might reduce protein aggregation in distant tissues in *Gba1b* mutants by decreasing production of dysregulated EVs that promote propagation of protein aggregates. To test this hypothesis, we examined knockdown of *neutral sphingomyelinase* (*nSMase*), encoding a lipid-modifying enzyme important for the formation and release of EVs [38,39]. nSMase hydrolyzes sphingomyelin, producing phosphocholine and ceramide. This enzyme has been implicated in multiple cellular functions, including inflammatory responses, reaction to lung and cardiac pathology, synaptic regulation, and release of EVs independent of ESCRT machinery [40]. We hypothesized that tissue-specific RNAi knockdown of *nSMase* in *Gba1b* mutants could reduce protein aggregation in distant tissues by reducing the production of dysregulated EVs promoting protein aggregation. However, if *Gba1b* mutant phenotypes are due to relative Cer depletion rather than accumulation of GlcCer, then *nSMase* knockdown might enhance the cell-autonomous protein aggregation in *Gba1b* mutants as Cer is known to trigger EV release [38]. We observed the latter, with increased accumulation of ubiquitinated proteins and Ref(2)P in thoraces (Fig 4A and 4C), suggesting that Cer depletion promotes cell-autonomous protein aggregation. However, we also observed a trend towards an increase in ubiquitinated proteins and Ref(2)P in the heads of *Gba1b* mutants with knockdown of *nSMase* expression in flight muscle (Fig 4B and 4D), suggesting that *nSMase* knockdown in muscle is not sufficient to reduce non-cell-autonomous spread of protein aggregates to other tissues, and may be enhancing non-cell-autonomous spread of protein aggregates. In contrast, knock down of *nSMase* in neuronal tissue did not modify Ref(2)P or ubiquitinated protein levels in the heads of *Gba1b* mutants (Fig 4E and 4F). These results suggest that nSMase-mediated release of EVs and/or influence on Cer metabolism may be important for the cell-autonomous regulation of protein aggregation in *Gba1b* mutant muscles but not neurons.

**Fig 5:**
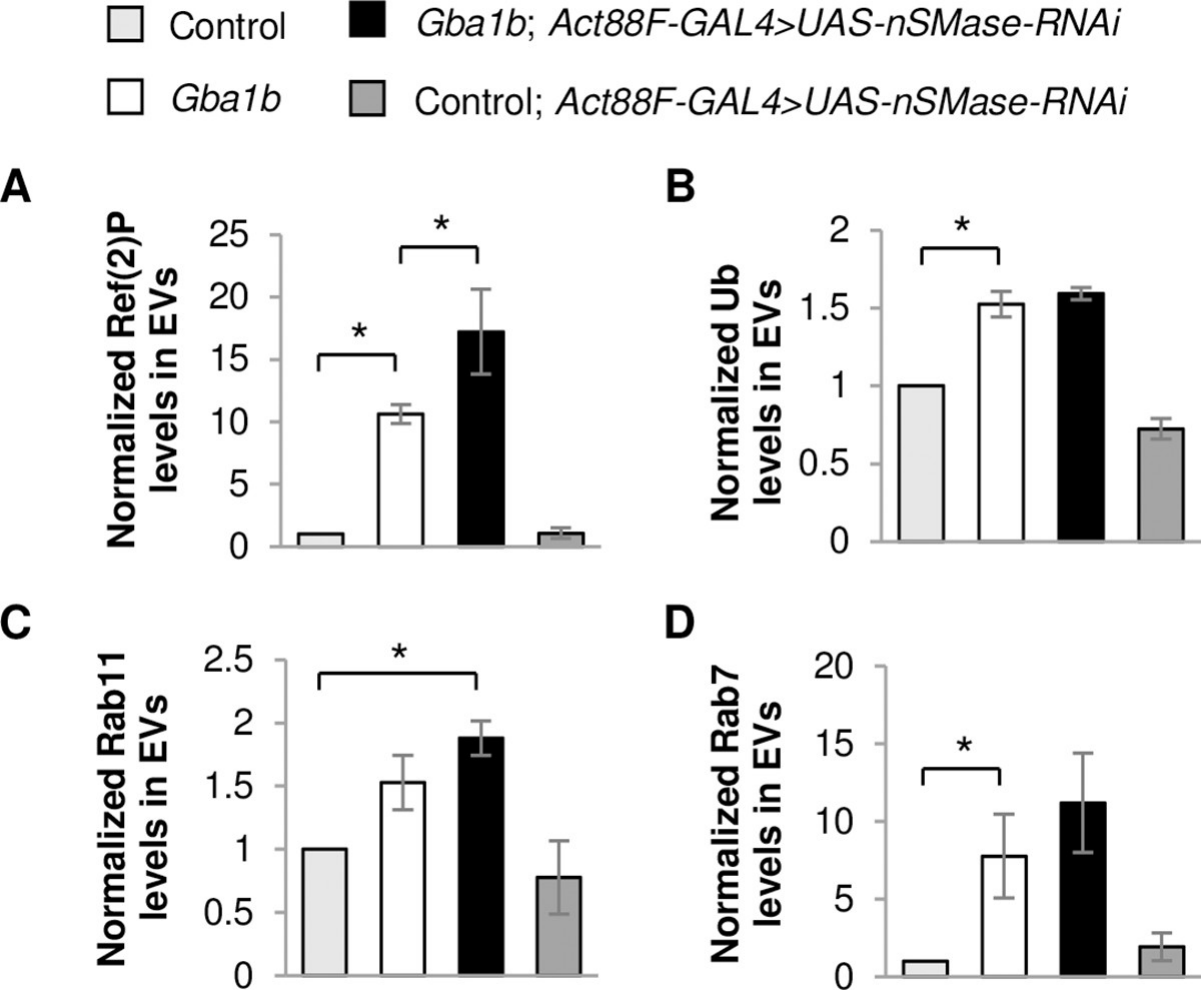
Muscle-specific knockdown of nSMase alters EV protein cargo. (A-D) *Neutral sphingomyelinase (nSMase)-*RNAi was expressed using the flight muscle driver *Act88F-GAL4* in *Gba1b* mutants and wildtype revertant controls. Isolated EVs from these flies were prepared in RIPA buffer. Quantification of western blot analysis of (A) Ref(2)P (one-way ANOVA: F(3,8) = 20.397, p < 0.001), (B) ubiquitin (F(3,8) = 55.25, p < 0.001), (C) Rab11 (F(3,8) = 6.713, p = 0.014), and (D) Rab7 (F(3,8) = 5.159, p = 0.028) in the EV fraction are shown. Results are normalized to control. At least 3 independent experiments were performed. Error bars represent SEM. *p < 0.05 by Student t-test. (Representative images are found in S1 Fig).

We next examined whether EV cargo is altered in *Gba1b* mutants with RNAi knockdown of *nSMase* in muscle. We found an even further increase in Ref(2)P in EVs isolated from *Gba1b* mutants with *nSMase* knocked down in muscle (Fig 5A). Rab11 which was elevated in *Gba1b* mutants was significantly increased compared to controls after knockdown of nSMase in the muscle of mutant flies (Fig 5C). There was no significant change in the levels of ubiquitinated proteins or Rab7, which were already increased in EVs from *Gba1b* mutants (Fig 5B and 5D). This suggests that nSMase may regulate the protein cargo and abundance of specific types of EVs.

**Fig 6:**
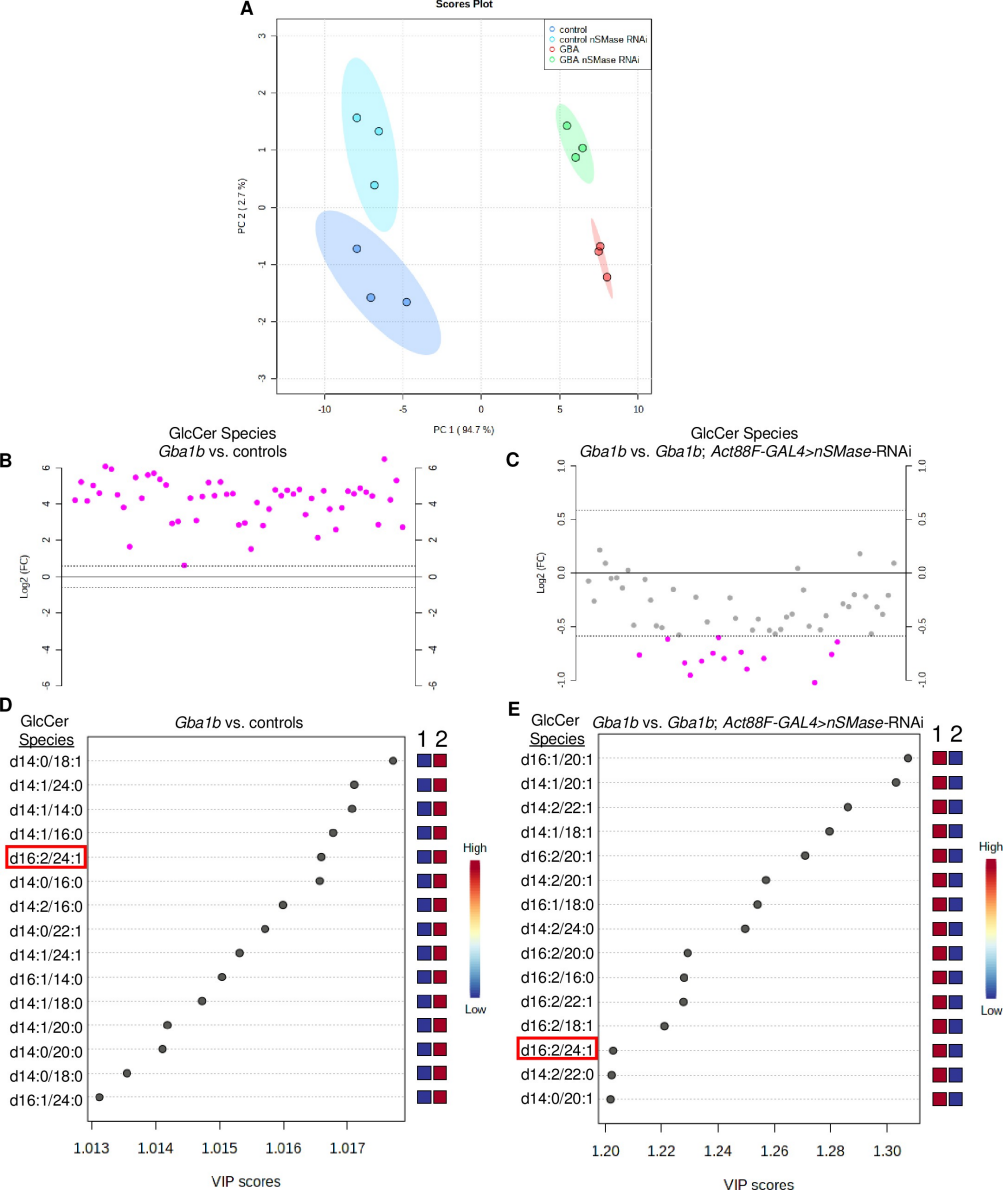
Muscle-specific knockdown of *nSMase* minimally decreases glucosylceramide levels, which are globally elevated in *Gba1b* mutants. Targeted lipidomic analysis of glucosylceramide (GlcCer) species in 10 day old whole flies of the specified genotypes. (A) Principal component analysis (PCA) scores plot of all detected GlcCer species in control, *Gba1b*, *Gba1b*; *Act88F-GAL4>nSMase-*RNAi, and control*; Act88F-GAL4>nSMase-*RNAi flies. Refer to S1 Data for significant compounds (in bold) contributing to PC1 and PC2, which account for 97.4% of the variance. Ovals indicate 95% confidence region. (B) GlcCer species plotted by fold change between *Gba1b* and controls. Pink circles represent GlcCer species above a threshold fold change of 1.5 (dotted line), FDR ≤0.05. Values are plotted on log scale so that both up-regulated and down-regulated GlcCer species can be plotted in a symmetrical way. (C) GlcCer species plotted by fold change in *Gba1b* versus *Gba1b*; *Act88F-GAL4>nSMase-*RNAi. Pink circles represent GlcCer species above a threshold of 1.5-fold change (dotted line), FDR ≤0.10. Values are plotted on log scale. (D) Top 15 significant GlcCer species identified by Partial Least Squares–Discriminant Analysis (PLS-DA) in *Gba1b* versus controls (R^2^ = 0.99, Q^2^ = 0.98, 2 components), ranked by VIP scores. Refer to S2B Fig for PLS-DA scores plot of GlcCer species in *Gba1b* versus control. Variable Importance in Projection (VIP) is a weighted sum of squares of the PLS loadings (S2 Data) taking into account the amount of explained Y-variation in each dimension. The colored boxes on the right indicate the relative concentrations of the corresponding GlcCer species in each genotype. The first column (1) is control; the second column (2) is *Gba1b*. (E) Top 15 significant GlcCer species identified by PLS-DA in *Gba1b* versus *Gba1b*; *Act88F-GAL4>nSMase-*RNAi as ranked by VIP score (R^2^ = 0.99, Q^2^ = 0.79, 2 components, refer to S2D Fig for scores plot). The colored boxes on the right indicate the relative concentrations of the corresponding GlcCer species in each genotype. The first column (1) is *Gba1b*; the second column (2) is *Gba1b*; *Act88F-GAL4>nSMase-*RNAi. The highlighted species GlcCer d16:2/24:1 is also found in the top 15 significant GlcCer species identified by PLS-DA for *Gba1b* versus controls (D).

**Fig 7:**
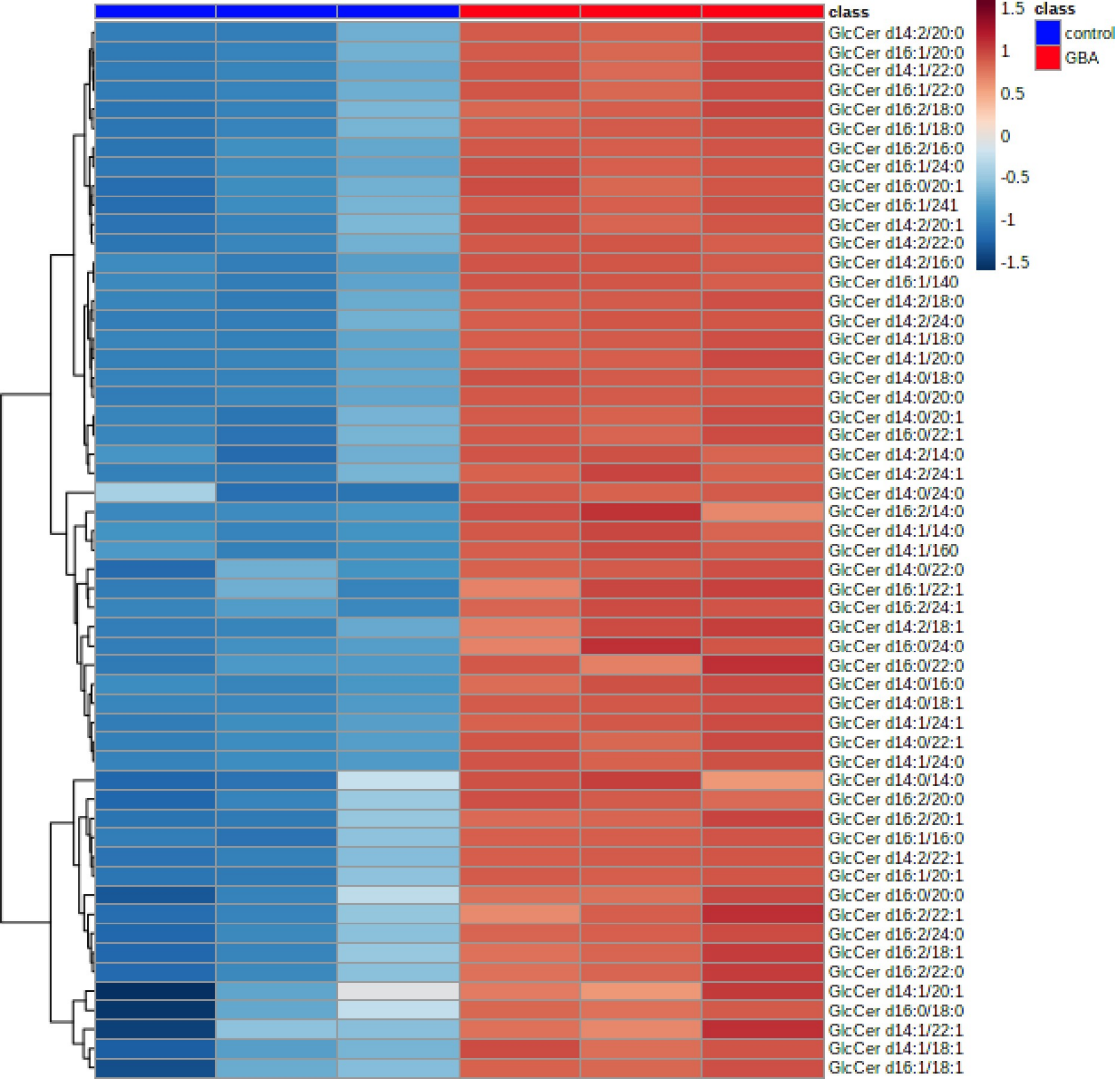
Glucosylceramide levels are globally elevated in *Gba1b* mutants compared to controls. Targeted lipidomic analysis of glucosylceramide (GlcCer) species in 10 day old whole flies. Clustering analysis represented by a heat map of all detected GlcCer species in control (columns 1–3) versus *Gba1b* (columns 4–6).

To investigate whether the observed effects of *nSMase*-RNAi on cell-autonomous protein aggregation and EV cargo are due to alterations in Cer metabolism, we performed targeted lipidomic analysis to determine Cer and GlcCer levels in *Gba1b* mutants and controls with and without *nSMase* knocked down in muscle. We detected 39 Cer and 55 GlcCer species by liquid chromatography-mass spectroscopy (LC-MS) from whole bodies of 10 day old flies. Principal component analysis (PCA) of GlcCer species in *Gba1b* mutants and controls with and without *nSMase* knocked down in muscle revealed 4 distinct groups which could be distinguished by 2 main principal components (PC) (Fig 6A). Most of the variance (94.7%) was explained by PC1, separating control and *Gba1b* mutants. The top 15 significant GlcCer species for the loadings for PC1 and PC2 are listed in S1 Data. We found the levels of all GlcCer species in *Gba1b* mutants to be significantly elevated, as highlighted by the heat map displaying clustering analysis for GlcCer species in *Gba1b* vs control (Fig 7). All the GlcCer species were increased in *Gba1b* mutants by a fold-change of ≥1.5 compared to controls (Fig 6B), and 31 (56%) GlcCer species were increased over 20-fold for a False Discovery Rate (FDR) ≤ 0.05 (Fig 6B and Table 1, and S3A Fig). Consistent with PCA results, the effect of *nSMase* knock down in muscle on GlcCer levels had a modest effect on both control and *Gba1b* mutants but tended to decrease GlcCer levels (Fig 6C and 6E). Only one GlcCer species decreased by over 2-fold in *Gba1b* mutants expressing *nSMase*-RNAi in muscle compared to *Gba1b* mutants, and 14 GlcCer species decreased by over 1.5-fold with FDR ≤ 0.10 (Fig 6C and Table 2 and S3B Fig). Partial Least Squares–Discriminant Analysis (PLS-DA), a supervised multivariate regression analysis, was also performed to identify key GlcCer species differing between *Gba1b* mutants and controls, and *Gba1b* mutants with *nSMase-*RNAi in muscle vs *Gba1b* mutants. PCA and PLS-DA analyses resulted in very similar group separation of *Gba1b* mutants versus controls and *Gba1b* mutants expressing *nSMase-*RNAi in muscle versus *Gba1b* mutants (S2 Fig). Only 1 GlcCer species (GlcCer d16:2/24:1) in the top 15 significant GlcCer species identified by PLS-DA in *Gba1b* mutants expressing *nSMase*-RNAi in muscle versus *Gba1b* mutants (R^2^ = .99, Q^2^ = 0.98) was also in the top 15 GlcCer species differing significantly between *Gba1b* mutants versus controls (R^2^ = 0.99, Q^2^ = 0.79) (Fig 6D and 6E).

**Table 1:** Top 15 significant GlcCer species by Volcano plot in *Gba1b* mutants vs controls.

Compound	Fold Change	Adjusted *p* value
GlcCer d14:1/16:0	66.81	9.68E-05
GlcCer d14:0/18:1	48.15	9.68E-05
GlcCer d14:1/14:0	36.95	9.68E-05
GlcCer d14:1/24:0	26.04	9.68E-05
GlcCer d14:0/16:0	22.73	9.68E-05
GlcCer d16:2/24:1	7.29	9.68E-05
GlcCer d14:2/16:0	32.38	1.22E-04
GlcCer d14:0/22:1	27.46	1.26E-04
GlcCer d14:1/24:1	26.45	1.38E-04
GlcCer d16:1/14:0	60.18	1.42E-04
GlcCer d14:1/18:0	51.59	1.49E-04
GlcCer d14:0/20:0	23.13	1.63E-04
GlcCer d14:1/20:0	21.91	1.63E-04
GlcCer d14:0/18:0	33.00	1.85E-04
GlcCer d16:1/24:0	39.11	2.00E-04

* adjusted for FDR ≤ 0.05

**Table 2:** Significant GlcCer species identified by Volcano plot in *Gba1b* mutants expressing *nSMase-*RNAi in muscle vs. *Gba1b* mutants.

Compound	Fold Change	Adjusted *p* value
GlcCer d14:1/20:1	0.52	0.013
GlcCer d16:1/20:1	0.58	0.013
GlcCer d14:2/22:1	0.60	0.025
GlcCer d14:1/18:1	0.59	0.026
GlcCer d16:2/20:1	0.54	0.029
GlcCer d16:1/18:0	0.58	0.034
GlcCer d16:2/22:1	0.49	0.041
GlcCer d16:2/18:1	0.56	0.042
GlcCer d14:0/20:1	0.60	0.047
GlcCer d14:1/20:0	0.66	0.058
GlcCer d16:1/18:1	0.57	0.095
GlcCer d16:1/16:0	0.65	0.095

* adjusted for FDR ≤ 0.10

**Fig 8:**
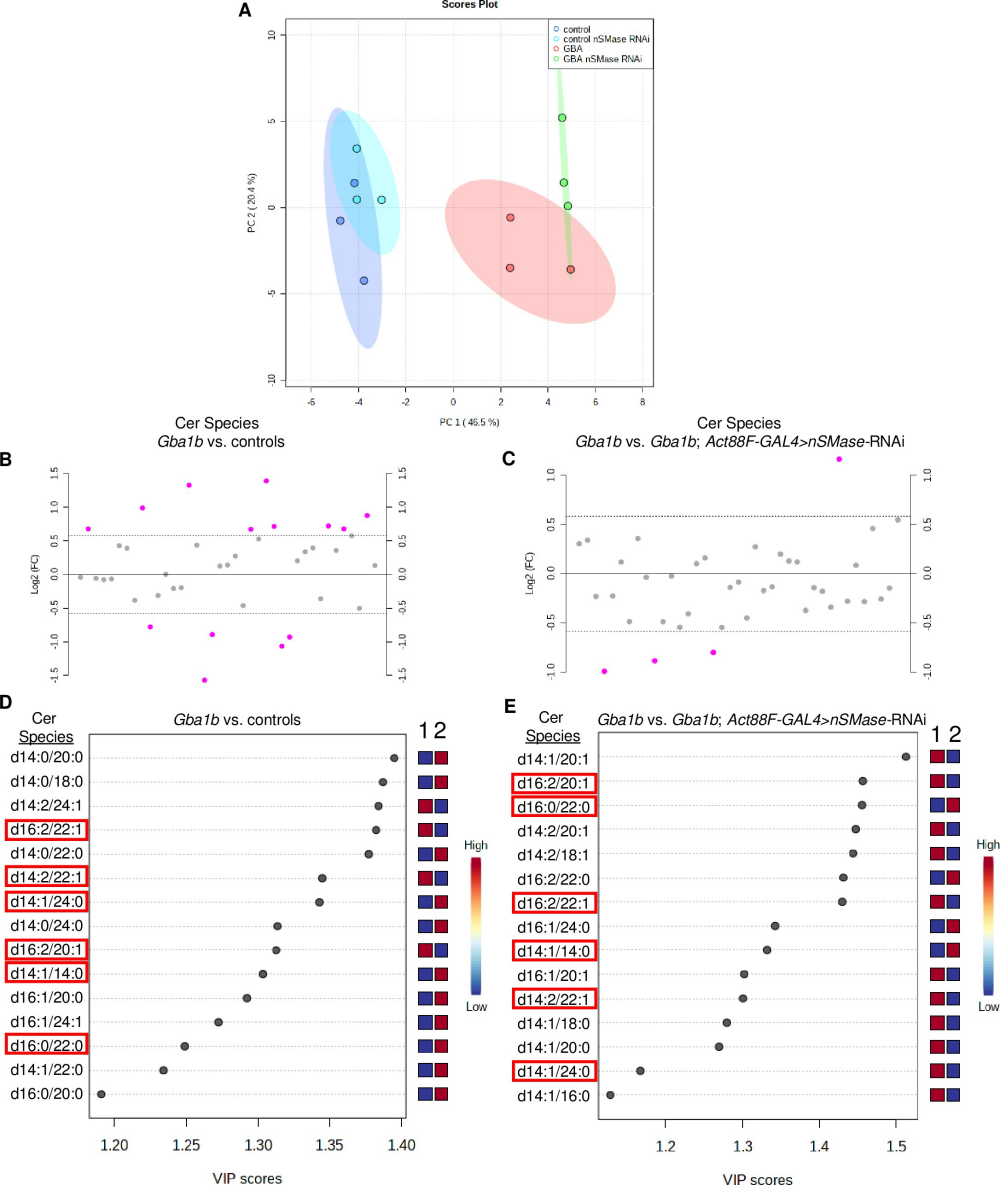
Muscle-specific knockdown of *nSMase* decreases a subset of ceramide species that are decreased in *Gba1b* mutants. Targeted lipidomic analysis of ceramide (Cer) species in 10 day old whole flies of the specified genotypes. (A) Principal component analysis of Cer species in control, *Gba1b*, *Gba1b*; *Act88F-GAL4>nSMase-*RNAi, and control*; Act88F-GAL4>nSMase-*RNAi flies. Ovals indicate 95% confidence region. (B) Cer species plotted by fold change in *Gba1b* versus controls. Pink circles represent Cer species above a 1.5-fold change threshold, FDR ≤0.10. Values are plotted on log scale so that both up-regulated and down-regulated Cer species can be plotted in a symmetrical way. (C) Cer species in *Gba1b*; *Act88F-GAL4>nSMase-*RNAi versus *Gba1b*, plotted by fold change. Pink circles represent Cer species above a 1.5-fold change threshold, FDR ≤0.10. Values are plotted on log scale. (D) Top 15 significant Cer species identified by Partial Least Squares–Discriminant Analysis (PLS-DA) in *Gba1b* versus controls (R^2^ = 0.99, Q^2^ = 0.80, 3 components), ranked by VIP score. The colored boxes on the right indicate the relative concentrations of the corresponding Cer species in each genotype. The first column (1) is control, the second column (2) is *Gba1b*. Refer to S4B Fig for PLS-DA scores plot of Cer species in *Gba1b* versus control. (E) Top 15 significant Cer species identified by PLS-DA in *Gba1b*; *Act88F-GAL4>nSMase-*RNAi versus *Gba1b* (R^2^ = 0.99, Q^2^ = 0.70, 2 components). The colored boxes on the right indicate the relative concentrations of the corresponding Cer species in each genotype. The first column (1) is *Gba1b*; the second column (2) is *Gba1b*; *Act88F-GAL4>nSMase-*RNAi. The highlighted species are also in the top 15 species identified by PLS-DA for *Gba1b* mutants versus controls (D).

**Fig 9:**
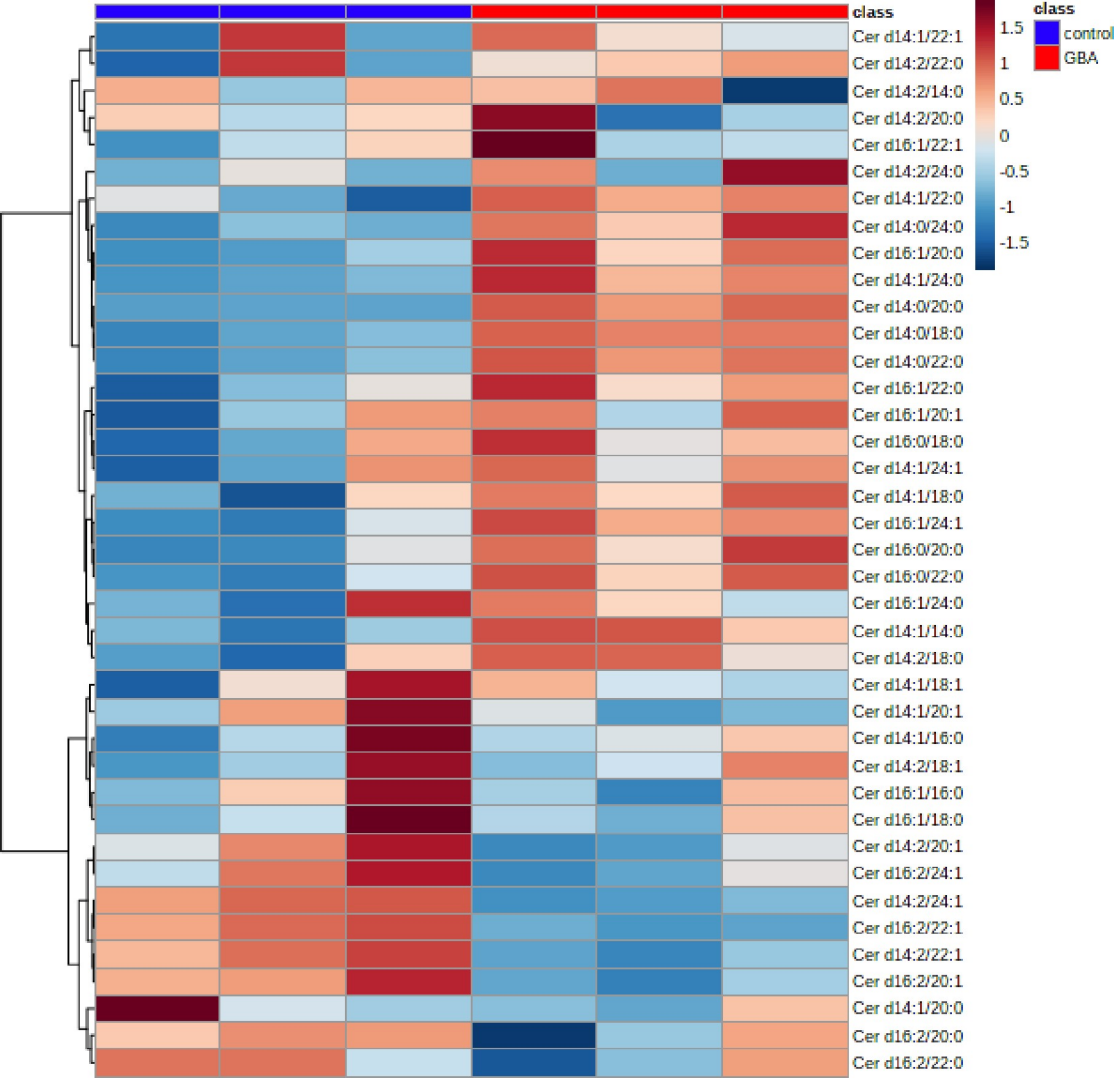
Most ceramide species are increased in *Gba1b* mutants compared to controls. Targeted lipidomic analysis of ceramide (Cer) species in 10 day old whole flies. Clustering analysis represented by a heat map of all detected Cer species in control (columns 1–3) versus *Gba1b* (columns 4–6).

PCA of Cer species in *Gba1b* mutants and controls with and without *nSMase* knocked down in muscle revealed a distinct separation between *Gba1b* mutants and controls, accounting for 46.5% variance, and distinct separation between *Gba1b* mutants expressing *nSMase-*RNAi in muscle versus *Gba1b* mutants, accounting for 20.4% variance (Fig 8A, significant Cer species for PCA loadings in S1 Data). Heat map derived from clustering analysis of Cer species in *Gba1b* mutants versus controls indicates that the majority of Cer species are increased in *Gba1b* mutants compared to controls (Fig 9), although there is a significant subset of Cer species that are decreased. Fold-change analysis revealed that the magnitude of alterations in Cer levels between *Gba1b* mutants and controls was much smaller in comparison to alterations of GlcCer species in *Gba1b* mutants versus controls (Fig 8B). Volcano plot with adjusted *p*-values for FDR ≤ 0.10 revealed 36% of Cer species had greater than 1.5-fold change in *Gba1b* mutants versus controls, with 4 Cer species decreased and 9 Cer species increased in *Gba1b* mutants versus controls (Table 3 and S5A Fig). PCA and PLS-DA analyses again resulted in similar discrimination between groups when comparing *Gba1b* mutants versus controls, and *Gba1b* mutants expressing *nSMase RNAi* in muscle versus *Gba1b* mutants, (S4 Fig and S1 and S2 Data). PLS-DA of Cer species in *Gba1b* mutants versus controls identified two Cer species (Cer d16:2/22:1, and Cer d14:2/22:1) that were significantly reduced in *Gba1b* mutants versus controls (R^2^ = 0.99, Q^2^ = 0.80) (Fig 8D and S6 Fig). These two Cer species were also within the top 15 loadings for PC1 in PCA of *Gba1b* mutants versus controls (S1 Data) and reduced by greater than 1.5 fold with adjusted *p*-value for FDR ≤ 0.10 in Volcano plot for *Gba1b* mutants versus controls (Table 3 and S5A Fig). Cer d16:2/22:1 and Cer d14:2/22:1 were found to be further significantly decreased by PLS-DA of *Gba1b* mutants expressing *nSMase*-RNAi in muscle versus *Gba1b* mutants (R^2^ = 0.99, Q^2^ = 0.70) (Fig 8E and Table 4), and again found to be significant contributors to PC1 in PCA of *Gba1b* mutants expressing *nSMase*-RNAi in muscle versus *Gba1b* mutants (S1 Data). These results confirm that the knockdown of nSMase expression by RNAi affects Cer metabolism, as the majority of Cer species in *Gba1b* mutants with *nSMase* knocked down in muscle decreased compared to *Gba1b* mutants, consistent with the predicted enzymatic effect of nSMase knockdown on Cer metabolism.

**Table 3:** Significant Cer species identified by Volcano plot in *Gba1b* mutants vs controls.

Compound	Fold Change	Adjusted *p* value*
Cer d14:0/20:0	2.506	0.004
Cer d14:2/24:1	0.478	0.004
Cer d14:0/18:0	1.984	0.004
Cer d16:2/22:1	0.525	0.004
Cer d14:0/22:0	2.620	0.005
Cer d14:2/22:1	0.337	0.017
Cer d14:1/24:0	1.649	0.017
Cer d16:2/20:1	0.538	0.028
Cer d14:0/24:0	1.600	0.028
Cer d14:1/14:0	1.600	0.031
Cer d16:1/24:1	1.836	0.043
Cer d14:1/22:0	1.593	0.060
Cer d16:0/20:0	1.641	0.087

* adjusted for FDR ≤ 0.10

**Table 4:** Significant Cer species identified by Volcano plot in *Gba1b* mutants expressing *nSMase-*RNAi in muscle vs *Gba1b* mutants.

Compound	Fold Change	Adjusted *p* value*
Cer d14:2/20:1	0.541	0.098
Cer d14:2/18:1	0.502	0.098
Cer d16:2/22:0	2.242	0.098

* adjusted for FDR ≤ 0.10

### Ectopically expressed GCase is trafficked within extracellular vesicles

**Fig 10:**
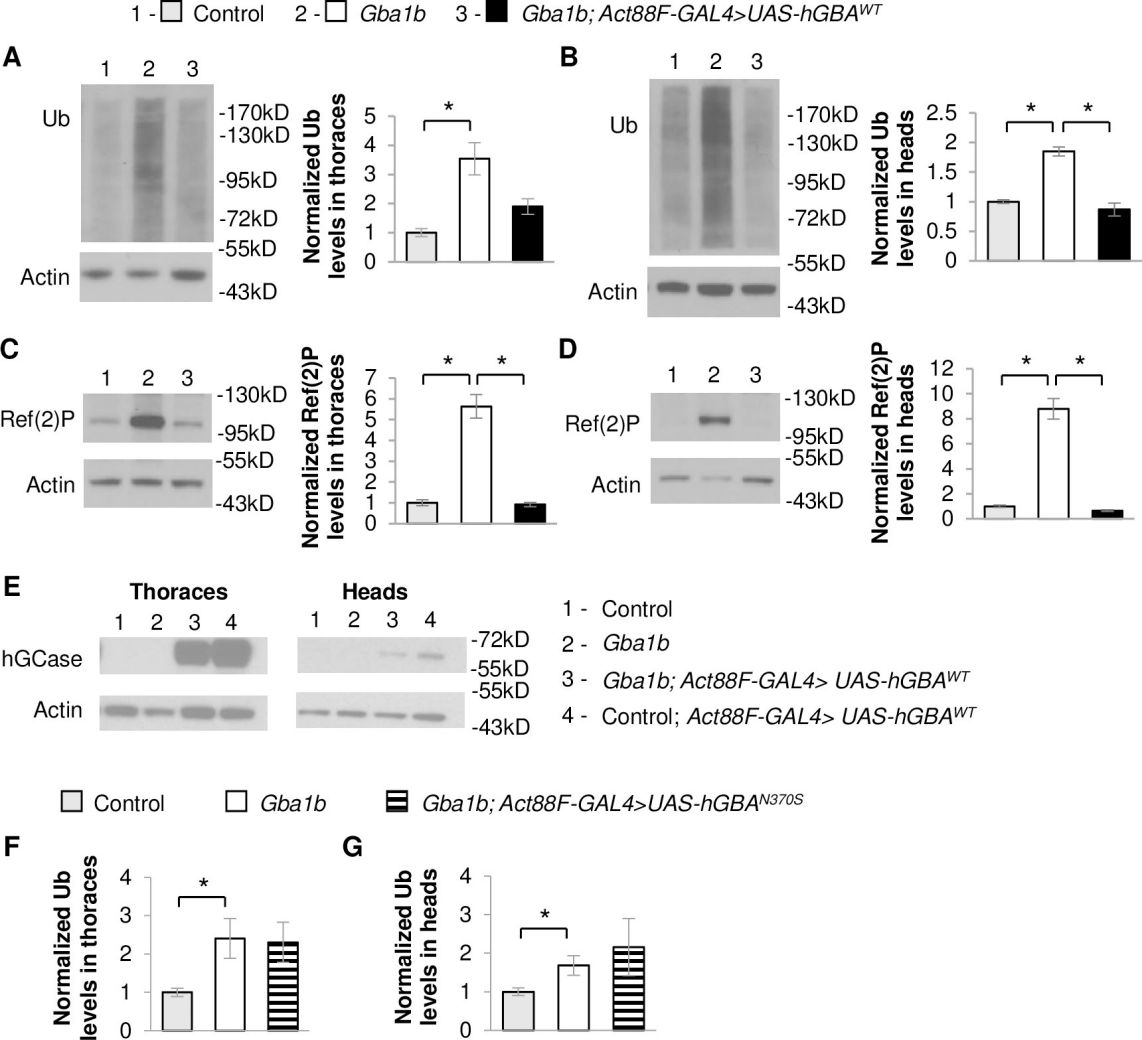
Muscle expression of human WT *GBA* suppresses protein aggregation in *Gba1b mutants*. (A-E) Using the flight muscle specific driver, *Act88F-GAL4*, wildtype (WT) human *GBA* (*hGBA*^*WT*^) was expressed in *Gba1b* mutant and WT revertant controls. Homogenates were prepared from fly heads and thoraces using 1% Triton X-100 lysis buffer. Western blot analysis was performed on the Triton X-100 insoluble proteins using antibodies to ubiquitin (Ub) and Actin, and on the soluble fractions using antibodies to Ref(2)P, Actin, and human glucocerebrosidase (hGCase). Representative images and quantification of ubiquitin in (A) thoraces (One-way ANOVA: F(2,6) = 19.949, p = 0.002) and (B) heads (F(2,6) = 24.854, p = 0.001) and Ref(2)P in (C) thoraces (F(2,6) = 10.609, p = 0.011) and (D) heads (F(2,6) = 23.297, p = 0.001) of *Gba1b* mutant flies with and without muscle expression of *hGBA*^*WT*^ are shown. Results are normalized to Actin and control. (E) Antibody detecting hGCase in the thoraces and heads of control and *Gba1b* mutant flies with and without muscle expression of *hGBA*^*WT*^. (F,G) Using *Act88F-GAL4*, mutant human *GBA* (*hGBA*^*N370S*^) was expressed in *Gba1b* mutant and controls. Western blot analysis was performed on the Triton X-100 insoluble proteins using antibodies to ubiquitin (Ub) and Actin. Quantification of ubiquitin in (F) thoraces (F(2,6) = 5.190, p = 0.049) and (G) heads (F(2,6) = 7.250, p = 0.025) of *Gba1b* mutant flies with and without muscle expression of *hGBA*^*N370S*^ are shown. Results are normalized to Actin and control. (Representative images are found in S7 Fig). At least 3 independent experiments were performed. Error bars represent SEM. *p < 0.05 by Student t-test.

**Fig 11:**
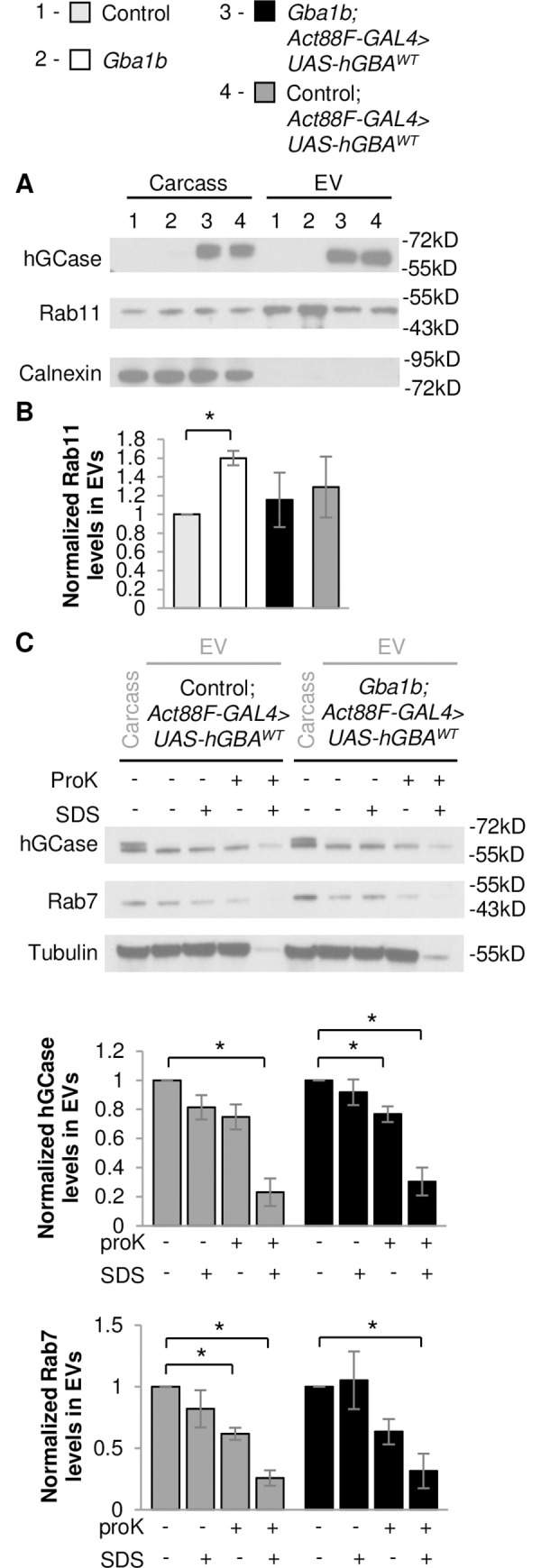
Ectopically expressed human GCase is trafficked within EVs. (A-B) Fly carcass homogenates and isolated extracellular vesicles (EVs) from *Gba1b* mutants and wildtype (WT) revertant controls with and without WT human *GBA* (*hGBA*^*WT*^*)* expressed in flight muscle using the *Act88F-GAL4* driver were prepared using RIPA buffer. Western blot analysis was performed using antibodies to human glucocerebrosidase (hGCase), Rab11, Rab7, and Tubulin. (A) Representative images of hGCase and Rab11 in carcass and isolated EVs from control and *Gba1b* mutant flies with and without muscle expression of *hGBA*^*WT*^ are shown. The blots were also probed with antibodies to Calnexin (Cnx99A) to confirm the purity of the EV samples. (B) Levels of Rab11 in the EV fraction are quantified and normalized to controls. (C) Representative images of hGCase, Rab7, and Tubulin in carcass and isolated EVs from control and *Gba1b* mutant flies with muscle expression of *hGBA*^*WT*^ with and without Proteinase K (ProK) and/or SDS exposure. Levels of hGCase (One-way ANOVA, control with *hGBA*^*WT*^ expression: (F(3,8) = 18.388, p < 0.001) and *Gba1b* mutants with *hGBA*^*WT*^ expression: (F(3,8) = 19.490, p < 0.001)) and Rab7 (control with *hGBA*^*WT*^ expression: (F(3,8) = 13.916, p = 0.002) and *Gba1b* mutants with *hGBA*^*WT*^ expression: (F(3,8) = 5.550, p = 0.023)) in the EV fraction are quantified. Results are normalized to EVs without ProK or SDS exposure. At least 3 independent experiments were performed. Error bars represent SEM. *p < 0.05 by Student t-test.

Our finding that *GBA* expression in muscle normalizes Ref(2)P levels in EVs suggests that non-cell-autonomous rescue is mediated at least in part by preventing spread of aggregated proteins trafficked by EVs. However, EV-mediated trafficking of GCase could also be another contributing factor to the non-cell-autonomous rescue of protein aggregation in *Gba1b* mutants. To test this possibility, we investigated whether ectopically expressed GCase travels to distant tissues via EVs. Because there are no antisera that recognize *Drosophila* GCase, we examined ectopic expression of human *GBA (hGBA)* in *Gba1b* mutants. We previously found ubiquitous expression of WT *hGBA* (*hGBA*^*WT*^) to be sufficient to partially rescue the shortened lifespan of *Gba1b* mutants [19]. Here, we found that expression of *hGBA*^*WT*^ in flight muscle also reduced ubiquitinated protein aggregation and Ref(2)P accumulation in *Gba1b* mutants in both thoraces and heads (Fig 10A–10D). EVs isolated from *Gba1b* mutants expressing *hGBA*^*WT*^ in flight muscle were found to have decreased Rab11 levels compared to *Gba1b* mutants (Fig 11A and 11B), confirming that human GCase (hGCase) can also revert alterations in EVs due to *dGba1b* deficiency. We also expressed mutant *hGBA* bearing the common N370S mutation (*hGBA*^*N370S*^) in muscle, which failed to reduce the increased ubiquitin levels in the thoraces and heads of *Gba1b* mutant flies (Fig 10F and 10G). These results support a functional equivalence between *Drosophila* GCase and hGCase in reducing the formation of protein aggregates. Interestingly, we were also able to detect hGCase in both the thoraces and heads of *Gba1b* mutants expressing *hGBA*^*WT*^ in flight muscle (Fig 10E), indicating that hGCase itself is able to travel to distant tissues. Furthermore, we detected hGCase in EVs isolated from hemolymph of flies expressing *hGBA*^*WT*^ in muscle (Fig 11A). To determine whether hGCase is localized within EVs or associated on the outer surface of EVs, we added Proteinase K to the isolated EV fraction. We found hGCase to be resistant to Proteinase K digestion of the EV fraction but degraded if EVs were first treated with a detergent to disrupt vesicular membranes before Proteinase K digestion (Fig 11C). These results indicate that GCase can be incorporated into EV cargo, and that WT GCase may be trafficked by EVs to distant tissues to reduce protein aggregation.

## Discussion

Many genetic influences of PD have now been identified, and much work has been focused on how these genes lead to protein aggregation through mechanisms such as protein misfolding and autophagy defects. However, none of these genes have been implicated in cell-to-cell spread of pathogenic protein aggregates, which closely correlates with clinical disease progression. Our proteomic analysis and non-cell-autonomous rescue of protein aggregation in *Gba1b* mutants has led us to hypothesize that *GBA* mutations may influence the rate of propagation of protein aggregates between neurons. Our work suggests a link between *GBA* mutations and faster spread of intracellular protein aggregates via a novel EV-mediated mechanism, possibly explaining the recent clinical finding that *GBA* mutations accelerate the progression of clinical disease. Using a *Drosophila* model of *GBA* deficiency that manifests accelerated protein aggregation, we found that expressing WT GCase in specific tissues of a *GBA*-deficient fly can not only rescue protein aggregation cell-autonomously and in distant tissues, but also rescue alterations in protein cargo observed in EVs isolated from *Gba1b* mutant hemolymph. Interestingly, ectopically expressed WT GCase itself was found within EVs of *GBA*-deficient flies, suggesting that the non-cell-autonomous rescue due to GCase expression is mediated by both reduction in aggregated proteins in EVs and trafficking of GCase via EVs to distant cells and tissues. Perturbing EV biogenesis through decreased expression of ESCRT-independent nSMase affected protein aggregation in local tissues in a tissue-dependent manner, and further decreased a subset of Cer species already reduced in *Gba1b* mutants. Interestingly, this subset of Cer species is known to be enriched in EV membranes [41]. Together, these findings suggest that mutations in *GBA* result in the accelerated spread of protein aggregates through changes in cellular lipid composition and dysregulation of proteins trafficked by EVs.

Although our model of *GBA* mutations promoting spread of protein aggregates via EVs is novel, the idea that proteostasis can be maintained in a non-cell-autonomous fashion is well supported in the literature. For example, in *C*. *elegans*, misfolded α- synuclein accumulating in endo-lysosomal vesicles was found to be transmitted from muscle to the hypodermis, a nearby tissue, for degradation [42]. It is possible that a non-cell-autonomous mechanism is necessary because certain tissues may be more efficient in reducing protein aggregation. This has been previously described, where overexpression of *FOXO* in *Drosophila* muscle decreased aging-related protein aggregates in muscle as well as brain and other distant tissues, but *FOXO* overexpression in adipose tissue was unable to prevent protein aggregation in muscle [43]. In our model, overexpressing *dGba1b* in *Drosophila* muscle or neuronal tissue prevented accumulation of protein aggregates throughout the organism, however overexpression of WT GCase in midgut and fat body did not significantly reduce protein aggregation in the brain (S8 Fig). These discrepancies could be due to tissue-specific biogenesis of EVs, which could depend on factors such as metabolic rate or endovesicular trafficking. Although *dGba1b* is expressed in all tissues, a second homologue of human *GBA1*, *dGba1a*, is expressed only in the midgut. Our *Gba1b* mutants retain ~25% expression of *dGba1a* [19]. Deficiency of *dGba1a* was found to extend lifespan [20] and does not result in significant accumulation of GlcCer [21], suggesting that there can be significant tissue-specific differences in function for GCase that could influence EV biogenesis.

Our unexpected results from perturbation of EV biogenesis suggest that the EV-mediated regulation of protein aggregation is tissue-specific and complex. Because an increase in EV-intrinsic proteins and alteration of protein cargo were observed in *Gba1b* mutants [23], we anticipated that genetic perturbations decreasing the biogenesis of EVs might rescue protein aggregation non-cell-autonomously by reducing the production of dysregulated EVs. However, decreased expression of ESCRT-independent *nSMase* in muscle did not rescue protein aggregation in heads, suggesting that a tissue-specific decrease in biogenesis of dysregulated EVs is not sufficient to reduce protein aggregation in the rest of the organism, and the cargo of EVs may need to be corrected to reduce spread of protein aggregation. In contrast, decreased expression of *nSMase* in the nervous system had no effect on protein aggregation in the head. This difference in outcome in perturbation of EV biogenesis in muscle and neurons could be due to cell-specific compensatory mechanisms or intrinsic metabolic demands and solicits further investigation.

A possible explanation for why decreased muscle expression of *nSMase* enhanced cell-autonomous protein aggregation and EV protein cargo alterations is that both GCase and nSMase enzymatically produce Cer. If GCase-deficient phenotypes are dependent on a relative reduction in Cer, decreased *nSMase* expression could exacerbate *Gba1b* mutant phenotypes. Indeed, lipidomic analysis of alterations in Cer metabolism due to *nSMase* knockdown revealed a further decrease in a subset of Cer species that were already significantly decreased in *Gba1b* mutants compared to controls. The further reduction in Cer species due to *nSMase* knockdown correlates with enhancement of cell-autonomous protein aggregation and EV cargo alterations, suggesting that accelerated protein aggregation in *Gba1b* mutants is mediated by Cer deficiency rather than GlcCer accumulation, as *nSMase* knockdown had a much more modest effect on the significantly increased levels of GlcCer species in *Gba1b* mutants compared to controls.

Cer has been implicated in the composition and biogenesis of EVs, and nSMase knockdown further altered EV cargo in *Gba1b* mutants, suggesting that decreased Cer levels may directly influence EV biogenesis in *Gba1b* mutants. However, Cer species were not globally decreased, suggesting that the regulation of Cer metabolism is complex and may be more dependent on specific Cer species. Interestingly, only 1 of the 9 Cer species significantly increased in *Gba1b* mutants versus controls had a monounsaturated fatty acyl group, while all 5 of the Cer species significantly decreased in *Gba1b* mutants versus controls had a monounsaturated fatty acyl group, suggesting *GBA* influences the metabolism of specific subset of Cer species that may be implicated in *Gba1b* mutant phenotypes. This subset of Cer species is enriched in species with long chain monounsaturated fatty acyl chains. Interestingly, lipids with monounsaturated fatty acyl groups are an abundant component in mammalian exosome membranes [40]. Investigating the alterations in lipid composition of EVs resulting from GCase deficiency and *nSMase* knockdown will be important in elucidating the role of Cer metabolism in *Gba1b* mutant phenotypes.

Our work suggests that GCase deficiency influences EV biogenesis to promote faster propagation of pathogenic protein aggregates throughout the tissues of an organism, which may be a compensatory response to cell-autonomous lysosomal stress. In the initial characterization of our *Drosophila GBA*-deficient model we found accelerated insoluble ubiquitinated protein aggregates, accumulation of Ref(2)P, and oligomerization of ectopically expressed human α-synuclein in *Gba1b* mutants, suggesting an impairment in lysosomal degradation [19]. A similar *GBA*-deficient *Drosophila* model also found evidence of lysosomal dysfunction, including enlarged lysosomes in *GBA*-deficient brains [20]. However, our proteomic analysis of *Gba1b* mutants did not support a profound impairment in autophagy, but instead suggested dysregulation of EVs with altered protein cargo which could be suppressed locally with knockdown of genes encoding ESCRT machinery important for EV biogenesis [23]. Based on these results, we believe that our initial observations of increased insoluble ubiquitinated proteins and Ref(2)P in *Gba1b* mutants are due to lysosomal stress. One possible explanation for our proteomic findings is that there may be a compensatory increase in EV biogenesis and packaging of autophagy substrates within EVs for discard outside of the cell in *Gba1b* mutants. Such an increase may have prevented us from detecting defects in autophagy. Upregulation of EV biogenesis may be cell-autonomously neuroprotective in the setting of lysosomal stress, particularly in aggregation-prone neurodegenerative diseases such as PD [44]. It was recently demonstrated in a human neuronal cell culture model of PD that inhibiting macroautophagy protects against α-synuclein-induced cell death by promoting the release of α-synuclein-containing EVs [45]. However, it remains possible that upregulating EV biogenesis may relieve lysosomal stress within cells containing aggregate-prone proteins, while simultaneously promoting the spread of protein aggregates between cells and throughout the organism.

Our work suggests a novel mechanism for *GBA* in reducing the spread of pathogenic protein aggregation from cell-to-cell via regulation of EV protein cargo, but many key questions remain. To better understand the progression of neurodegenerative diseases, we must uncover the mechanisms by which GCase deficiency alters EV protein content and biogenesis, identify the specific changes in EVs facilitating propagation of pathogenic protein aggregates, and determine how these changes influence recipient cells internalizing dysregulated EVs. GCase is a critical enzyme in ceramide metabolism, hydrolyzing glucosylceramide into glucose and ceramide. Ceramides are a key component of EV membranes, and alterations in ceramide metabolism due to GCase deficiency may directly influence EV biogenesis and protein cargo trafficked via EVs. Further studies using this *Drosophila* model and mammalian cell culture models should better elucidate how GCase deficiency alters the protein cargo of EVs to induce propagation of pathogenic protein aggregates, as well as whether endogenous GCase is enzymatically functional when trafficked to distant tissues via EVs. Understanding this mechanism could have broad implications in understanding the pathogenesis of aggregate-prone neurodegenerative diseases and reveal new therapeutic targets to slow or halt disease progression.

## Materials and methods

### *Drosophila* strains and culture

Fly stocks were maintained on standard cornmeal-molasses food at 25°C. The *Gba1b* homozygous null mutant (*Gba1b*^*ΔTT*^), isogenic control (*Gba1b*^*+*^*)*, and *UAS-dGba1b* alleles have been previously described [19]. All other strains and alleles were obtained from the Bloomington Stock Center: *elav-GAL4* (458); *Act88F-GAL4* (38459); *DMef-GAL4* (27390); *UAS-nSMase-RNAi* (36759); *Gba1b*^*MB03039*^*(23602)*. In Figs 4–7, we used the following genotypes for the experiments involving the *nSMase*-RNAi transgene: control = *Gba1b*^*+*^*/Gba1b*^*MB03039*^; *Gba1b* mutant = *Gba1b*^*ΔTT*^*/Gba1b*^*MB03039*^. This combination of *Gba1b* mutant alleles, which we used for ease of recombination with the transgene, produce the same biochemical abnormalities found in *Gba1b*^*ΔTT*^ homozygotes [23].

### Lifespan analysis

Longevity assays were conducted at 25°C. Groups of 10–20 age-matched flies were collected at 0–24 hours old and transferred to fresh standard food every 2–3 days. The number of dead flies was recorded during each transfer. Transfers were continued until all flies died. Kaplan-Meier lifespan curves were generated using Stata (StataCorp, College Station, TX), and analyzed by Cox proportional hazard models for statistically significant differences in survival between tested genotypes.

### Preparation of Triton-soluble and insoluble fractions

Tissues from 10-day-old flies (6 females and 6 males per sample) were homogenized in Triton lysis buffer (50 mM Tris-HCl pH 7.4, 1% Triton X-100, 150 mM NaCl, 1 mM EDTA), and then spun at 15,000 x *g* for 20 min. The detergent-soluble supernatant was collected and mixed with an equal volume of 2x Laemmli buffer (4% SDS, 20% glycerol, 120 mM Tris-Cl pH 6.8, 0.02% bromophenol blue, 2% β-mercaptoethanol), and the same buffer was used to resuspend the Triton-insoluble pellet. All samples were boiled for 10 minutes. The Triton-insoluble protein extracts were then cleared of debris by centrifugation at 15,000 x *g* for 10 minutes, followed by collection of the supernatant. At least three independent experiments were performed.

### Western blotting

Proteins were separated by SDS-PAGE on 4%-20% MOPS-acrylamide gels (GenScript Express Plus, M42012) and electrophoretically transferred onto Immobilon PVDF membranes (Fisher, IPVH00010). Immunodetection was performed using the iBind Flex Western Device (Thermo Fisher, SLF2000). Antibodies were used at the following concentrations: 1:10,000 mouse anti-Actin (Chemicon/Bioscience Research Reagents, MAB1501), 1:250 mouse anti-Rab11 (BD Transduction Laboratories, 610657), 1:50 mouse anti-Rab7 (DSHB Rab7), 1:200 rabbit anti-Ref(2)P (Abcam, ab178440), 1:500 mouse anti-ubiquitin (Santa Cruz, sc-8017), 1:10,000 mouse anti-tubulin (DSHB 12G10), 1:1500 rabbit anti-human GBA1 (Sigma, G4171), 1:800 mouse anti-Cnx99A (DSHB, Cnx99A 6-2-1). HRP secondary antibodies were used as follows: 1:500 to 1:1000 anti-mouse (BioRad, 170–6516) and 1:500 to 1:1000 anti-rabbit (BioRad, 172–1019). Signal was detected using Pierce ECL Western Blotting Substrate (Fisher, 32106). Densitometry measurements of the western blot images were performed using Fiji software [46]. (See uncropped western blot images in S4 Data.) For homogenates, signal was normalized either to Actin or Ponceau S [47,48]. Normalized western blot data were log-transformed when necessary to stabilize variance before means were compared using one-way ANOVA and Student *t*-test. Each experiment was performed at least three times.

### Immunohistochemistry

10-day-old adult brains were dissected in cold Schneider’s *Drosophila* medium (Thermo Fisher, 21720), and fixed in 4% paraformaldehyde/PBS for 30 min. Samples were washed in 0.1% Triton X-100/PBS. Fixed brains were stained with 1:200 mouse anti-poly Ubiquitin FK2 (Enzo, BML-PW8810), then anti-mouse Alexa 488 (1:200) and were mounted using ProLong Gold anti-fade medium (Molecular Probes, P10144).

### Extraction of hemolymph and preparation of EV fractions

Hemolymph was obtained from 30 flies (15 males and 15 females, 10 to 11 days old) per sample. All flies were frozen with liquid nitrogen and decapitated by vortexing. Frozen flies were placed in a 1.7-mL tube containing a volume of PBS scaled to the number of flies used (2 μL/fly) and thawed for 5 min at room temperature. The tubes were then centrifuged at 5000 x *g* for 5 min at 4°C, after which the extracted hemolymph (supernatant) was centrifuged for 30 min at 10,000 x *g* at 4°C to remove cell debris and the cell-free supernatant was collected. The supernatant was then filtered with Ultrafree 0.22 μm spin filters (Fisher, UFC30GV0S) and centrifuged at 3000 x *g* for 5 min at 4°C; this was the EV fraction. In order to obtain whole-fly homogenate from the same animals used for collection of hemolymph, the bodies from four flies (2 males and 2 females) were homogenized in RIPA buffer (150mM NaCl, 1% Nonidet P-40, 0.5% Sodium deoxycholate, 0.1% SDS, 50mM Tris pH 8, diluted 1:1 with ddH_2_0), centrifuged at 10,000 x *g* at 4°C for 5 min, and then the supernatant was transferred to a new tube. An equal volume of 2x Laemmli buffer (4% SDS, 20% glycerol, 120 mM Tris-Cl pH 6.8, 0.02% bromophenol blue, 2% β-mercaptoethanol) was added to the EV fractions and also to the whole-fly protein homogenates, and all samples were boiled for 10 min and then stored at −80°C. The experiment was repeated at least three times.

To determine if proteins were contained within EVs, before adding the Laemmli buffer, the EV fractions were incubated with PBS for 5 min at room temperature and then 0.5 μg/μL Proteinase K was added for 30 min at 4°C to digest external proteins or with 1% sodium dodecyl sulfate (SDS) for 5 min at room temperature and then 0.5 μg/μL Proteinase K was added for 30 min at 4°C to disrupt vesicular membranes and digest proteins. After incubation, Laemmli buffer was added and samples were boiled preparing them for western blotting.

### Lipidomic analysis

Fly tissue samples were prepared for LC-MS analysis by adding an equal volume of zirconium oxide beads to each sample of fly tissue (10 male and 10 female whole 10-day old flies). 200 μL water was added to each sample and homogenized using a Next Advance Bullet Blender at 4°C. 100 μL of homogenate was set aside for each sample for protein quantification. 50 μL of the isotope mix (Lipidyzer internal standard 1 μM d9-CER and 0.5 μM d9-HCER), 575 μL methyl tert-butyl ether (MTBE), and 160 μL methanol were added to the remaining 100 ul of each sample. All samples were vortexed and shaken for 30 minutes at room temperature at a 500 RPM setting. An additional 200 μL of water were added. The samples were centrifuged for 3 minutes at 12,000 x g at room temperature. The upper layers were transferred into glass culture tubes using glass pipettes. A second extraction was performed by adding 300 μL MTBE, 100 μL methanol, and 100 μL water. The samples were shaken for 5 minutes and centrifuged at the same settings as before. The upper layers were combined into the respective glass culture tubes and dried under a gentle stream of nitrogen. The samples were reconstituted in 250 μL of mobile phase B (60% acetonitrile, 40% isopropyl alcohol, 0.2% formic acid), and transferred to LC vials with inserts for LC-MS analysis.

Lipids were analyzed using a Shimadzu Nexera X2 HPLC (Shimadzu, Japan) coupled to an AB Sciex QTRAP 5500 hybrid triple quadrupole/linear ion trap mass spectrometer (AB Sciex LLC, USA) operating in positive electrospray ionization (ESI) mode. 39 ceramides and 55 glucosylceramides were monitored by multiple-reaction-mode (MRM) in a single batch run.

Lipidomic data were exported as csv files, grouped according to *Drosophila* strain and culture, and placed into MetaboAnalyst 4.0 web server (https://www.metaboanalyst.ca) for data processing and statistical analysis [49]. (See all lipidomic data in S3 Data.) Data was normalized by mass per sample, log-transformed and auto-scaled. The data were evaluated by univariate analysis methods (Fold Change analysis, T-tests, and Volcano plot) and multivariate analysis methods (principal component analysis and partial least square–discriminant analysis), and hierarchical clustering analysis. The False Discovery Rate (FDR), based on the Benjamini-Hochberg procedure, was set to <0.1 and used to select important Cer and GlcCer species. The FDR-adjusted p-value of <0.10 was used to control the number of false positives for multiple comparisons.

## Supporting information

S1 Fig Representative images for Fig 5. (A-D) *Neutral sphingomyelinase (nSMase)-*RNAi was expressed using the flight muscle driver *Act88F-GAL4* in *Gba1b* mutants and wildtype revertant controls. Isolated EVs from these flies were prepared in RIPA buffer. Representative images of (A) Ref(2)P, (B) ubiquitin, (C) Rab11, and (D) Rab7 in the EV fraction are shown.
(TIF)

S2 Fig Scores plots for PCA and PLS-DA for GlcCer species. (A) PCA scores plot of GlcCer species in *Gba1b* versus control flies. Ovals indicate 95% confidence region. Refer to S1 Data for significant compounds (in bold) contributing to PC1 and PC2, which account for 99.2% of the variance. (B) PLS-DA scores plot for PC1 and 2 for analysis of GlcCer species in *Gba1b* versus control flies (R^2^ = 0.99, Q^2^ = 0.98, 2 components). Ovals indicate 95% confidence region. Refer to S2 Data for significant compounds (in bold) contributing to PC1 and PC2. (C) PCA scores plot of GlcCer species in *Gba1b*; *Act88F-GAL4>nSMase-*RNAi versus *Gba1b* flies. Ovals indicate 95% confidence region. Refer to S1 Data for significant compounds (in bold) contributing to PC1 and PC2. (D) PLS-DA scores plot for analysis of GlcCer species in *Gba1b*; *Act88F-GAL4>nSMase-*RNAi versus *Gba1b* flies (R^2^ = 0.99, Q^2^ = 0.80, 2 components). Ovals indicate 95% confidence region. Refer to S2 Data for significant compounds (in bold) contributing to PC1 and PC2.
(TIF)

S3 Fig (A) Volcano plot of GlcCer species in *Gba1b* mutants versus controls with fold-change threshold set at 20 (vertical dotted lines) on the x-axis and t-test threshold of 0.05 (horizontal dotted line) on the y-axis. Pink circles represent GlcCer species above both thresholds. Note both fold changes and *p*-values are log transformed. The further its position away from the (0,0), the more significant the GlcCer species is. (B) Volcano plot with fold change threshold 1.5 on the x-axis and t-test threshold 0.1 on the y-axis. The pink circles represent GlcCer species above the threshold. Note both fold changes and *p* values are log transformed.
(TIF)

S4 Fig Scores plots for PCA and PLS-DA for Cer species. (A) PCA scores plot of Cer species in *Gba1b* versus control flies. Ovals indicate 95% confidence region. Refer to S1 Data for significant compounds (in bold) contributing to PC1 and PC2, which account for 99.2% of the variance. (B) PLS-DA scores plot for analysis of Cer species in *Gba1b* versus control flies (R^2^ = 0.99, Q^2^ = 0.80, 3 components). Ovals indicate 95% confidence region. Refer to S2 Data for significant compounds (in bold) contributing to PC1 and PC2. (C) PCA scores plot of Cer species in *Gba1b*; *Act88F-GAL4>nSMase-*RNAi versus *Gba1b* flies. Ovals indicate 95% confidence region. Refer to S1 Data for significant compounds (in bold) contributing to PC1 and PC2. (D) PLS-DA scores plot for analysis of GlcCer species in *Gba1b*; *Act88F-GAL4>nSMase-*RNAi versus *Gba1b* flies (R^2^ = 0.99, Q^2^ = 0.70, 2 components). Ovals indicate 95% confidence region. Refer to S2 Data for significant compounds (in bold) contributing to PC1 and PC2.
(TIF)

S5 Fig (A) Volcano plot of Cer species in *Gba1b* mutants versus controls with fold-change threshold 1.5 on the x-axis and t-tests threshold 0.1 on the y-axis. Pink circles represent Cer species above both thresholds. Note both fold changes and *p* values are log transformed. The further its position away from the (0,0), the more significant the Cer species is. (B) Volcano plot with fold change threshold 1.5 on the x-axis and t-test threshold 0.1 on the y-axis. Pink circles represent Cer species above both thresholds. Note both fold changes and *p* values are log transformed. The further its position away from the (0,0), the more significant the feature is.
(TIF)

S6 Fig Box plots of normalized values of Cer species that were significantly altered in both *Gba1b* vs controls, and *Gba1b*; *Act88F-GAL4>nSMase-*RNAi vs *Gba1b*, as determined by VIP score from PLS-DA, and within top 15 species of PC1 loadings for PCA of *Gba1b* vs control and *Gba1b*; *Act88F-GAL4>nSMase-*RNAi vs *Gba1b*. (TIF)

S7 Fig Representative images for Fig 8F and 8G. Using *Act88F-GAL4*, mutant human *GBA* (*hGBA*^*N370S*^) was expressed in *Gba1b* mutant and WT revertant controls. Homogenates were prepared from fly heads and thoraces using 1% Triton X-100 lysis buffer. Western blot analysis was performed on the Triton X-100 insoluble proteins using antibodies to ubiquitin (Ub) and Actin. Representative images of ubiquitin in (A) thoraces and (B) heads of *Gba1b* mutant flies with and without muscle expression of *hGBA*^*N370S*^ are shown.
(TIF)

S8 Fig Midgut and fat body expression of *dGba1b* does not rescue protein aggregation in *Gba1b* mutants. (A) Using the midgut driver *Npc1b-GAL4*, wildtype *dGba1b* was expressed in *Gba1b* mutants and wildtype revertant controls. Homogenates were prepared from fly heads using 1% Triton X-100 lysis buffer. Western blot analysis was performed on the Triton X-100 insoluble proteins using antibodies to ubiquitin (Ub) and Actin. Quantification of Ub in (A) heads of control and *Gba1b* mutants with and without midgut expression of *dGba1b* are shown. (B) Quantification of Ub in the heads of flies with and without *dGba1b* expression in fat body using the *Lsp2-GAL4* driver. Results are normalized to Actin and control. At least 3 independent experiments were performed. Error bars represent SEM. *p < 0.05 by Student t-test.
(TIF)

S1 Table Summary of Lifespans in Fig 1D. (PDF)

S2 Table Summary of Lifespans in Fig 2E. (PDF)

S1 Data PCA loadings. (XLSX)

S2 Data PLS-DA loadings. (XLSX)

S3 Data Targeted lipidomic results. (XLSX)

S4 Data All uncropped western blots. (PDF)
