# Supplementary figures and images for: Glucocerebrosidase reduces the spread of protein aggregation in a Drosophila melanogaster model of neurodegeneration by regulating proteins trafficked by extracellular vesicles
Source: PLoS Genet. 2021 Feb 4;17(2):e1008859. doi: 10.1371/journal.pgen.1008859 (PMC7888665; doi:10.1371/journal.pgen.1008859)

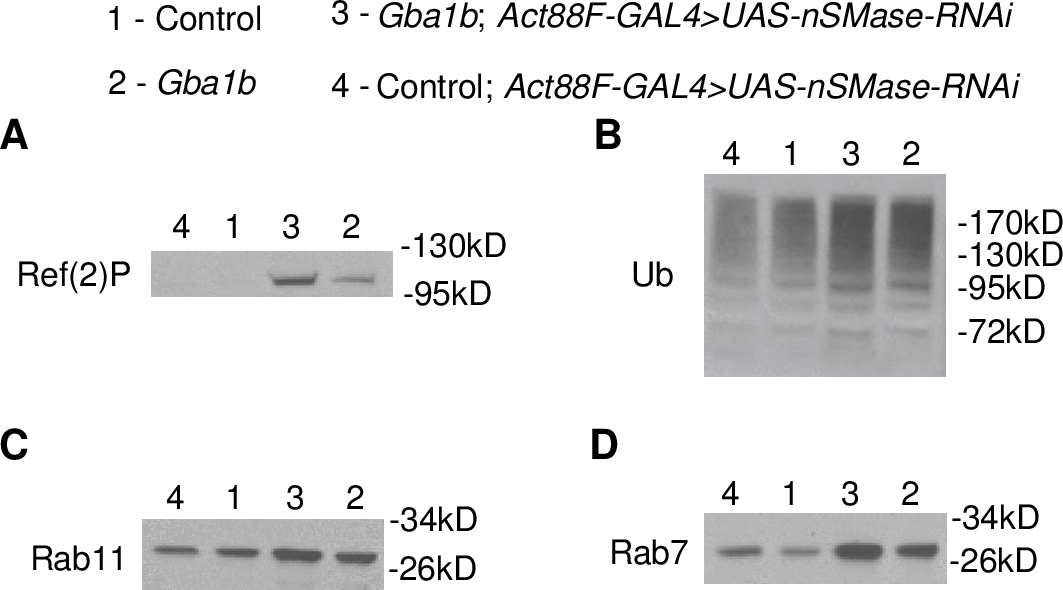

Supplement: S1 Fig — (A-D) Neutral sphingomyelinase (nSMase)-RNAi was expressed using the flight muscle driver Act88F-GAL4 in Gba1b mutants and wildtype revertant controls. Isolated EVs from these flies were prepared in RIPA buffer. Representative images of (A) Ref(2)P, (B) ubiquitin, (C) Rab11, and (D) Rab7 in the EV fraction are shown. (TIF) [file pgen.1008859.s001.tif]

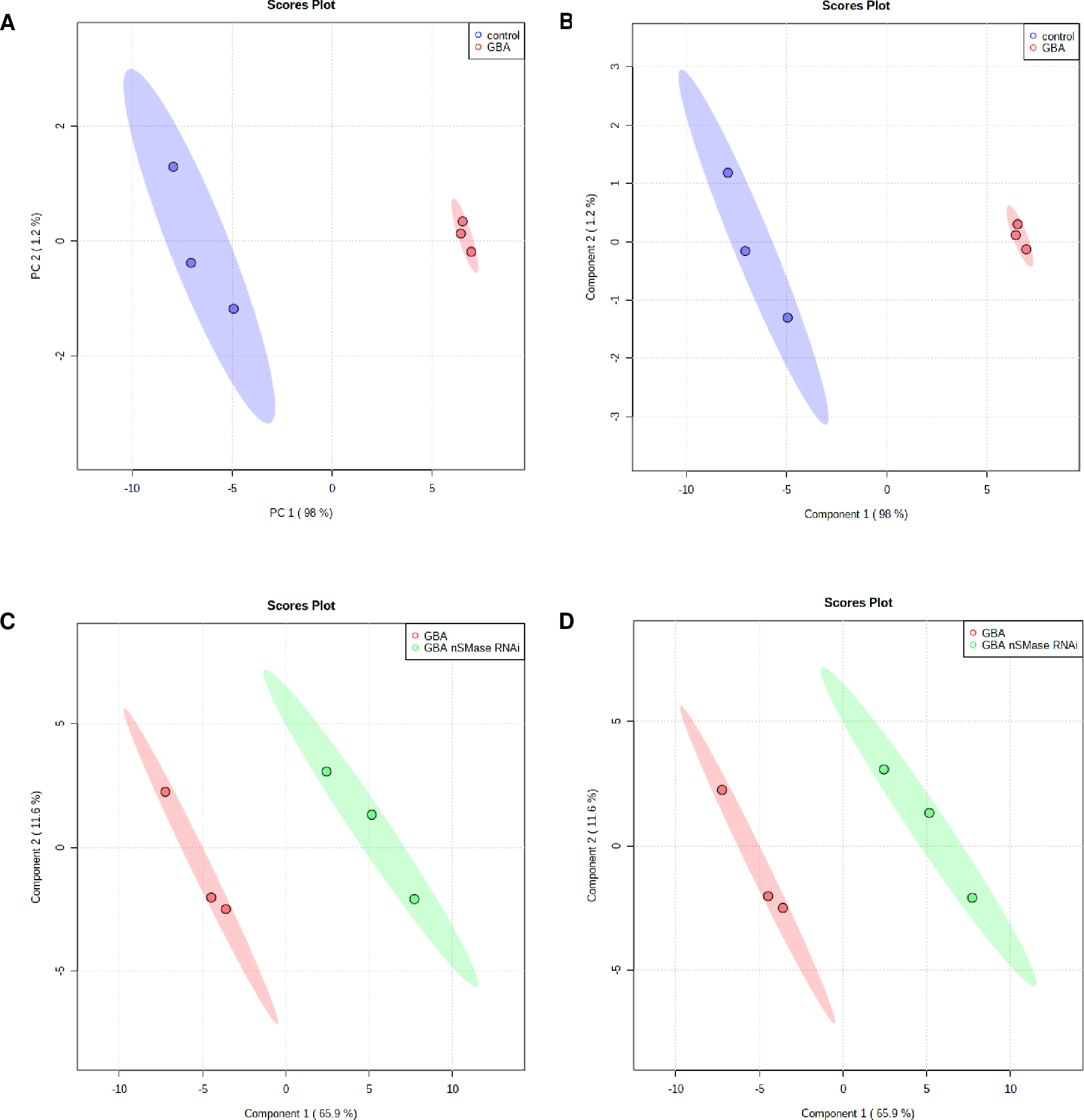

Supplement: S2 Fig — (A) PCA scores plot of GlcCer species in Gba1b versus control flies. Ovals indicate 95% confidence region. Refer to S1 Data for significant compounds (in bold) contributing to PC1 and PC2, which account for 99.2% of the variance. (B) PLS-DA scores plot for PC1 and 2 for analysis of GlcCer species in Gba1b versus control flies (R2 = 0.99, Q2 = 0.98, 2 components). Ovals indicate 95% confidence region. Refer to S2 Data for significant compounds (in bold) contributing to PC1 and PC2. (C) PCA scores plot of GlcCer species in Gba1b; Act88F-GAL4>nSMase-RNAi versus Gba1b flies. Ovals indicate 95% confidence region. Refer to S1 Data for significant compounds (in bold) contributing to PC1 and PC2. (D) PLS-DA scores plot for analysis of GlcCer species in Gba1b; Act88F-GAL4>nSMase-RNAi versus Gba1b flies (R2 = 0.99, Q2 = 0.80, 2 components). Ovals indicate 95% confidence region. Refer to S2 Data for significant compounds (in bold) contributing to PC1 and PC2. (TIF) [file pgen.1008859.s002.tif]

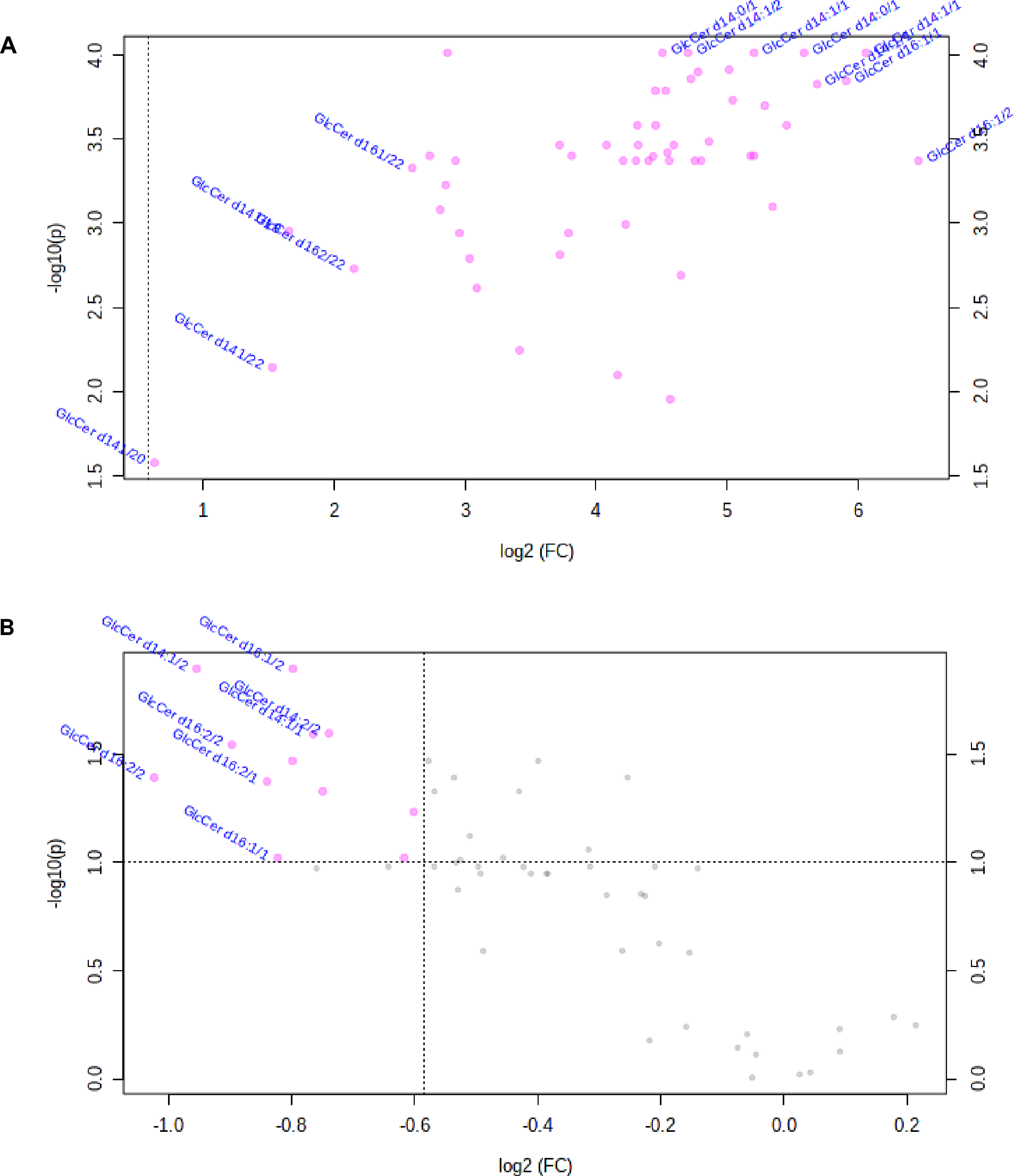

Supplement: S3 Fig — (A) Volcano plot of GlcCer species in Gba1b mutants versus controls with fold-change threshold set at 20 (vertical dotted lines) on the x-axis and t-test threshold of 0.05 (horizontal dotted line) on the y-axis. Pink circles represent GlcCer species above both thresholds. Note both fold changes and p-values are log transformed. The further its position away from the (0,0), the more significant the GlcCer species is. (B) Volcano plot with fold change threshold 1.5 on the x-axis and t-test threshold 0.1 on the y-axis. The pink circles represent GlcCer species above the threshold. Note both fold changes and p values are log transformed. (TIF) [file pgen.1008859.s003.tif]

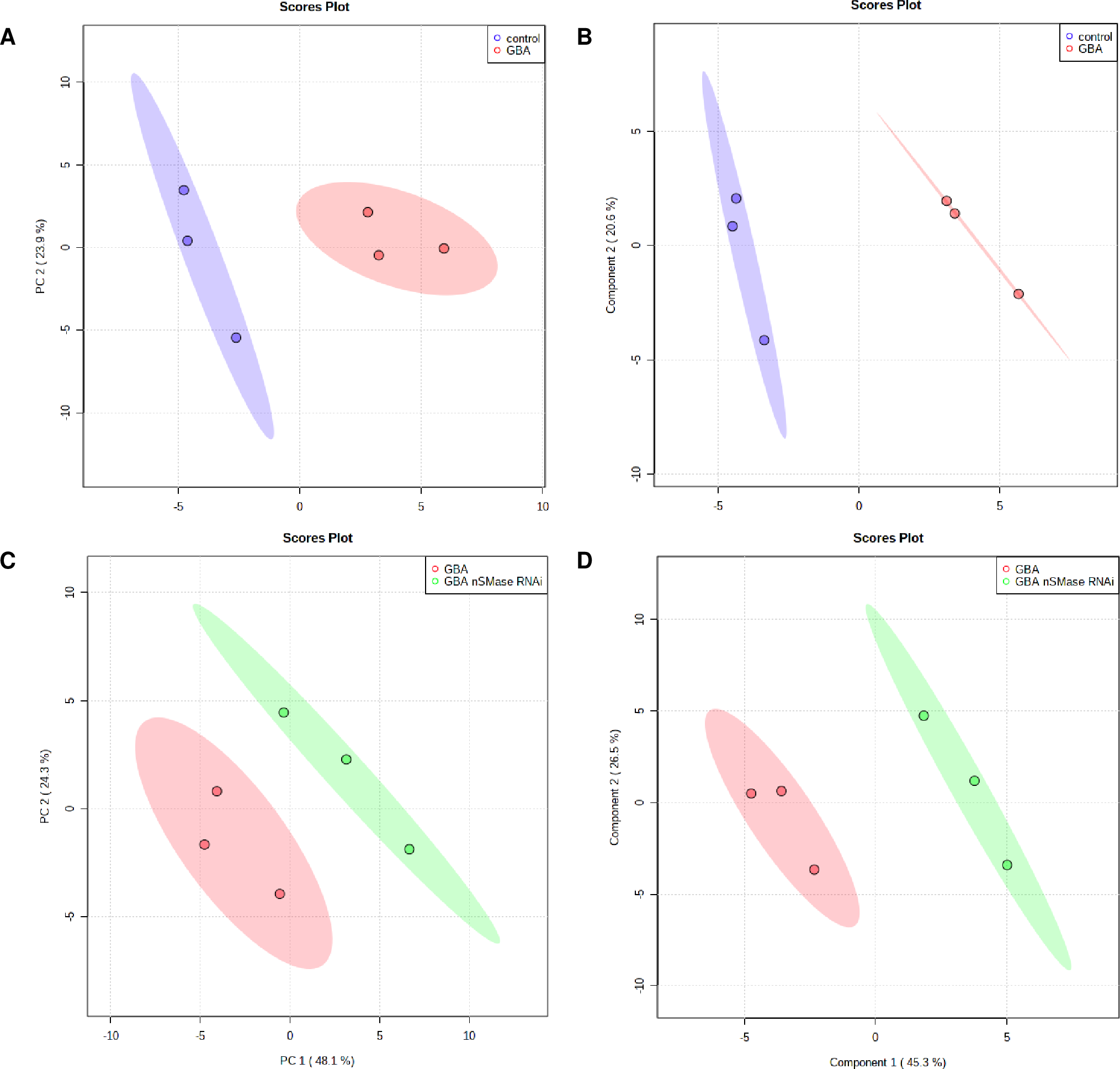

Supplement: S4 Fig — (A) PCA scores plot of Cer species in Gba1b versus control flies. Ovals indicate 95% confidence region. Refer to S1 Data for significant compounds (in bold) contributing to PC1 and PC2, which account for 99.2% of the variance. (B) PLS-DA scores plot for analysis of Cer species in Gba1b versus control flies (R2 = 0.99, Q2 = 0.80, 3 components). Ovals indicate 95% confidence region. Refer to S2 Data for significant compounds (in bold) contributing to PC1 and PC2. (C) PCA scores plot of Cer species in Gba1b; Act88F-GAL4>nSMase-RNAi versus Gba1b flies. Ovals indicate 95% confidence region. Refer to S1 Data for significant compounds (in bold) contributing to PC1 and PC2. (D) PLS-DA scores plot for analysis of GlcCer species in Gba1b; Act88F-GAL4>nSMase-RNAi versus Gba1b flies (R2 = 0.99, Q2 = 0.70, 2 components). Ovals indicate 95% confidence region. Refer to S2 Data for significant compounds (in bold) contributing to PC1 and PC2. (TIF) [file pgen.1008859.s004.tif]

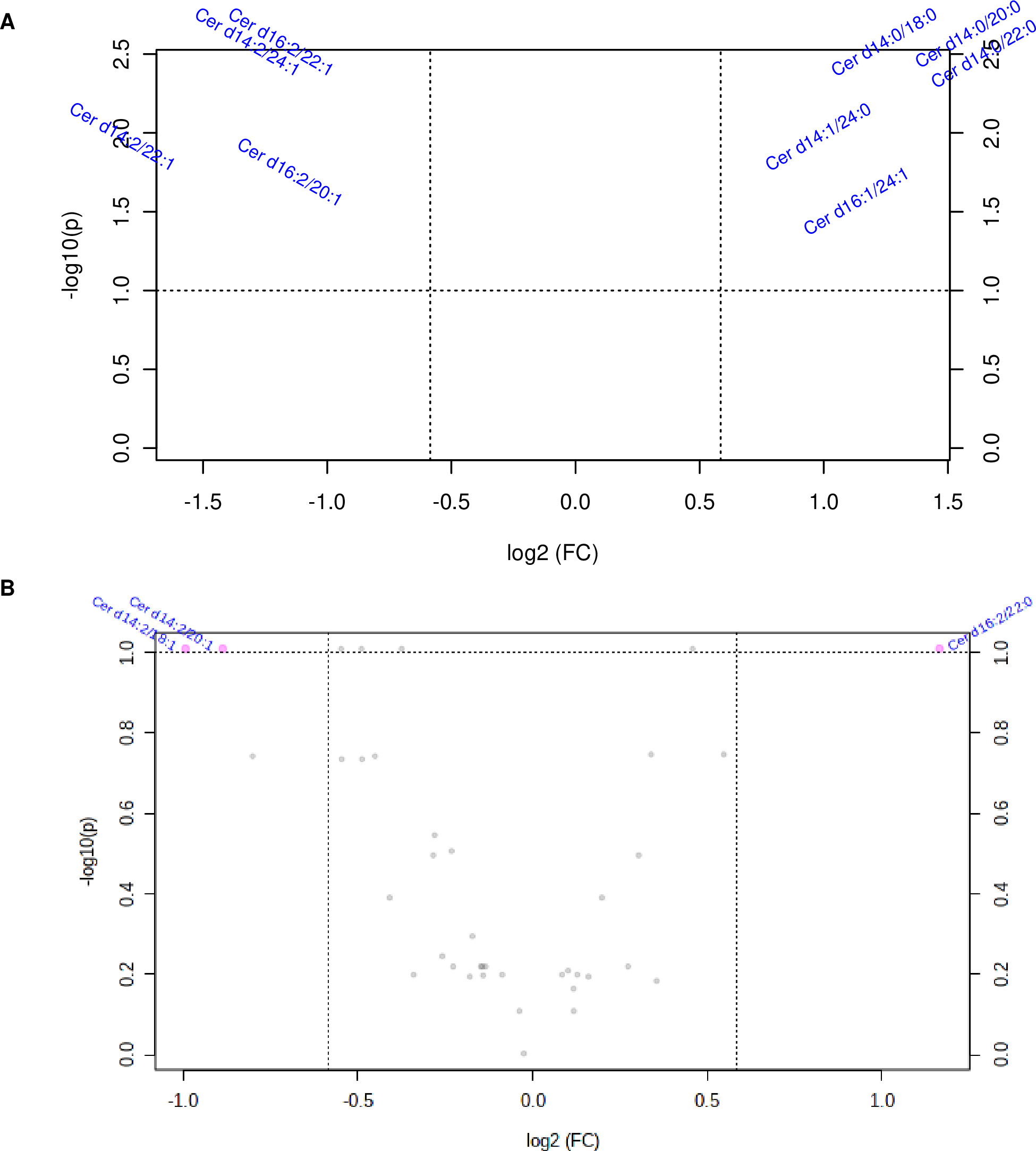

Supplement: S5 Fig — (A) Volcano plot of Cer species in Gba1b mutants versus controls with fold-change threshold 1.5 on the x-axis and t-tests threshold 0.1 on the y-axis. Pink circles represent Cer species above both thresholds. Note both fold changes and p values are log transformed. The further its position away from the (0,0), the more significant the Cer species is. (B) Volcano plot with fold change threshold 1.5 on the x-axis and t-test threshold 0.1 on the y-axis. Pink circles represent Cer species above both thresholds. Note both fold changes and p values are log transformed. The further its position away from the (0,0), the more significant the feature is. (TIF) [file pgen.1008859.s005.tif]

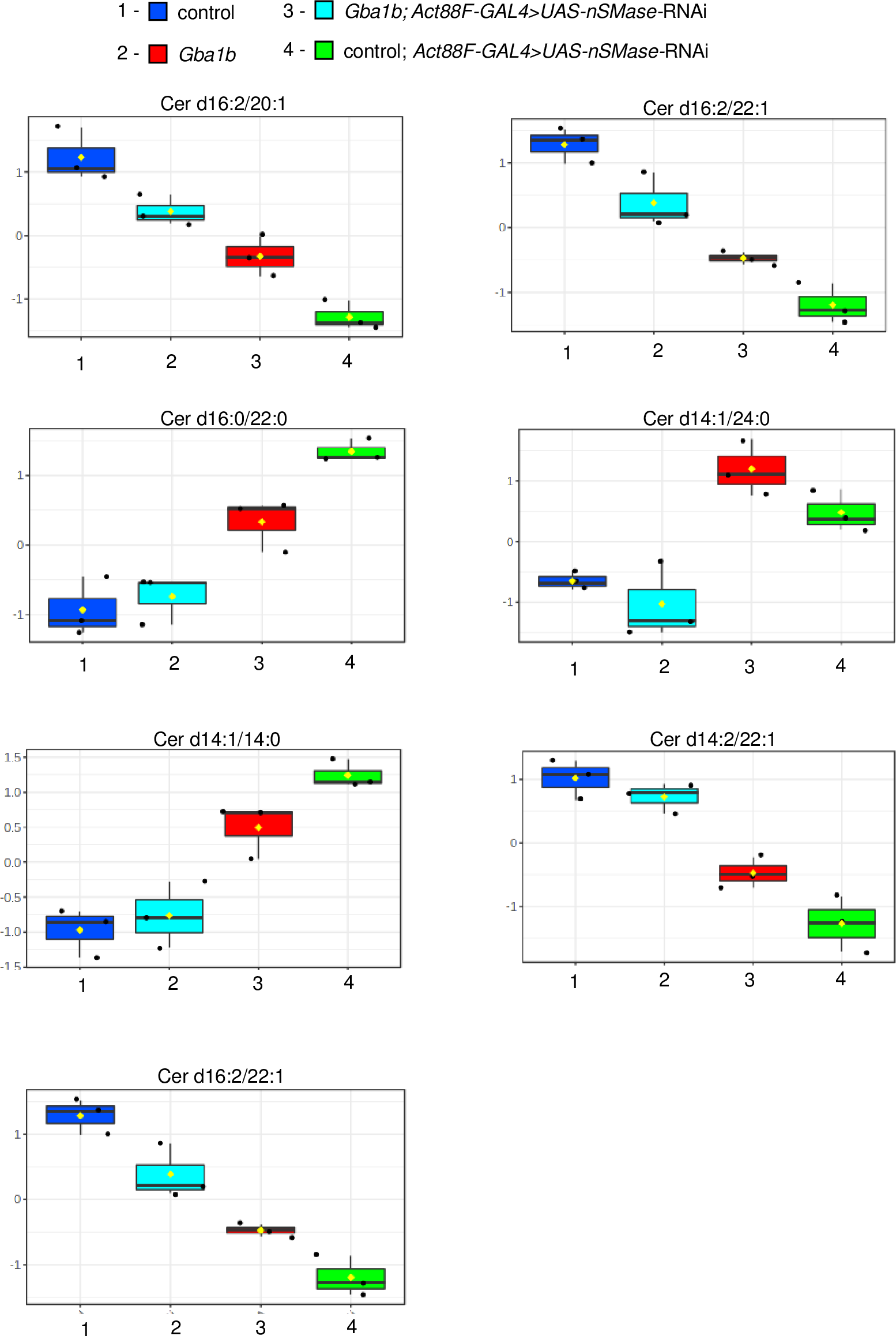

Supplement: S6 Fig — (TIF) [file pgen.1008859.s006.tif]

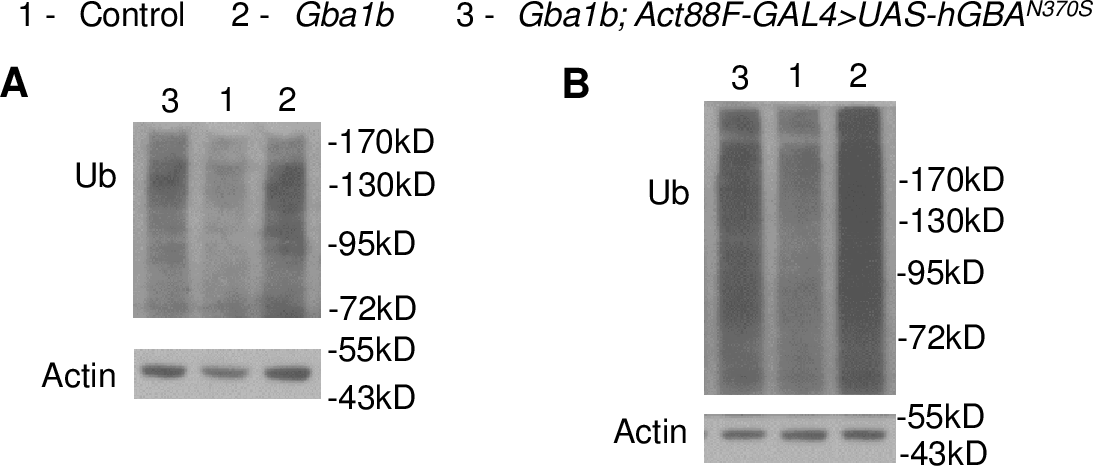

Supplement: S7 Fig — Using Act88F-GAL4, mutant human GBA (hGBAN370S) was expressed in Gba1b mutant and WT revertant controls. Homogenates were prepared from fly heads and thoraces using 1% Triton X-100 lysis buffer. Western blot analysis was performed on the Triton X-100 insoluble proteins using antibodies to ubiquitin (Ub) and Actin. Representative images of ubiquitin in (A) thoraces and (B) heads of Gba1b mutant flies with and without muscle expression of hGBAN370S are shown. (TIF) [file pgen.1008859.s007.tif]

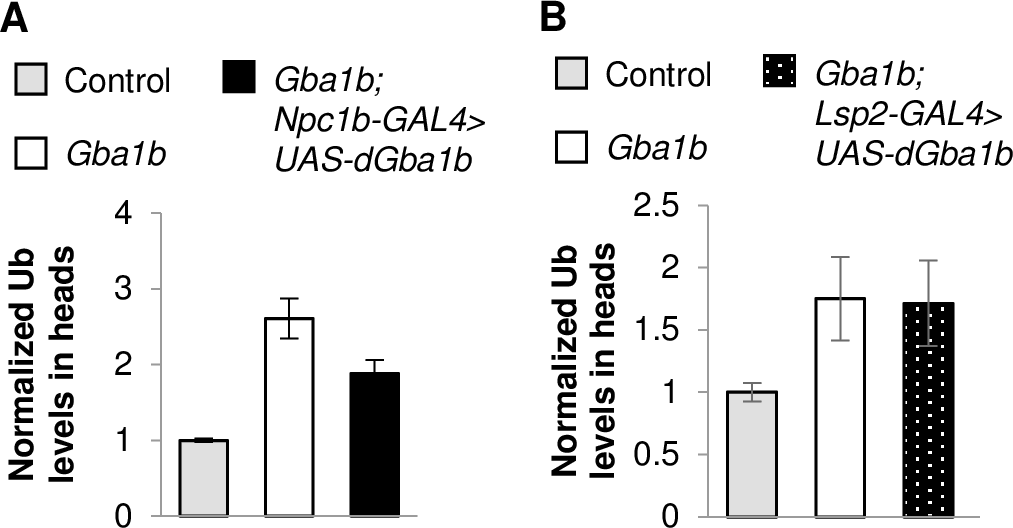

Supplement: S8 Fig — (A) Using the midgut driver Npc1b-GAL4, wildtype dGba1b was expressed in Gba1b mutants and wildtype revertant controls. Homogenates were prepared from fly heads using 1% Triton X-100 lysis buffer. Western blot analysis was performed on the Triton X-100 insoluble proteins using antibodies to ubiquitin (Ub) and Actin. Quantification of Ub in (A) heads of control and Gba1b mutants with and without midgut expression of dGba1b are shown. (B) Quantification of Ub in the heads of flies with and without dGba1b expression in fat body using the Lsp2-GAL4 driver. Results are normalized to Actin and control. At least 3 independent experiments were performed. Error bars represent SEM. *p < 0.05 by Student t-test. (TIF) [file pgen.1008859.s008.tif]
