# Supplementary material for: Glucocerebrosidase reduces the spread of protein aggregation in a Drosophila melanogaster model of neurodegeneration by regulating proteins trafficked by extracellular vesicles
Source: PLoS Genet. 2021 Feb 4;17(2):e1008859. doi: 10.1371/journal.pgen.1008859 (PMC7888665; doi:10.1371/journal.pgen.1008859)
Supplement: S1 Table — (PDF) [file pgen.1008859.s009.pdf]

S1 Table. Summary of Lifespans in Fig 1D

**Genotype**	***N***	**Mean survival (days)**	**95% Confidence Interval**	***p*** *
Control	416	60	61,64	< 0.0001
Gba1b	535	35	34,35	0.0001
Act88F-GAL4>UAS-Gba1b; control	409	73	71,75	< 0.0001
Act88F-GAL4>UAS-Gba1b; Gba1b	512	71	72,76	< 0.0001

**p* values determined by Cox proportional hazards model to test for equality of survival curves among all genotypes.
