# Supplementary material for: Glucocerebrosidase reduces the spread of protein aggregation in a Drosophila melanogaster model of neurodegeneration by regulating proteins trafficked by extracellular vesicles
Source: PLoS Genet. 2021 Feb 4;17(2):e1008859. doi: 10.1371/journal.pgen.1008859 (PMC7888665; doi:10.1371/journal.pgen.1008859)
Supplement: S2 Table — (PDF) [file pgen.1008859.s010.pdf]

S2 Table. Summary of Lifespans in Fig 2E

**Genotype**	***N***	**Mean survival (days)**	**95% Confidence Interval**	***p******
Control	392	60	59,62	< 0.0001
Gba1b	370	29	29,29	0.0019
Elav-GAL4>UAS-Gba1b; control	519	58	57,59	< 0.0001
Elav-GAL4>UAS-Gba1b; Gba1b	503	44	43,45	< 0.0001

*p values determined by Cox proportional hazards model to test for equality of survival curves among all genotypes.
