# Supplementary material for: Glucocerebrosidase reduces the spread of protein aggregation in a Drosophila melanogaster model of neurodegeneration by regulating proteins trafficked by extracellular vesicles
Source: PLoS Genet. 2021 Feb 4;17(2):e1008859. doi: 10.1371/journal.pgen.1008859 (PMC7888665; doi:10.1371/journal.pgen.1008859)

S4 Data – Western Blots

Fig. 1A

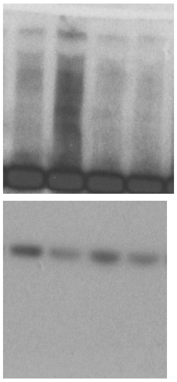

Fig. 1B

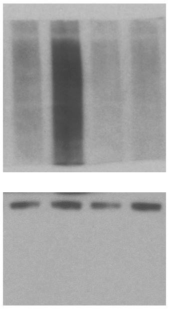

Fig. 1E

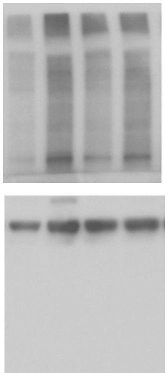

Fig. 1F

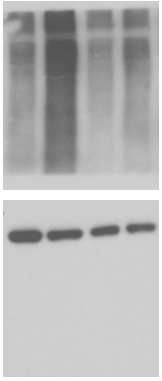

Fig. 1G

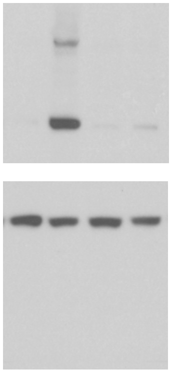

Fig. 1H

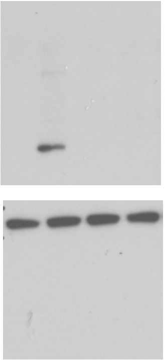

Fig. 2A

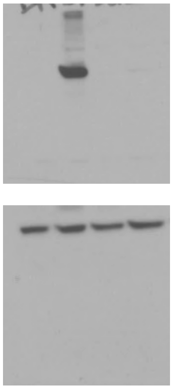

Fig. 2B

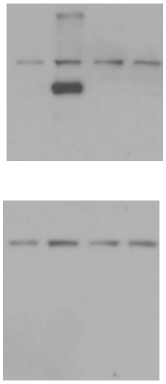

Fig. 2C

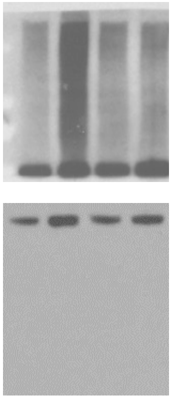

Fig. 2D

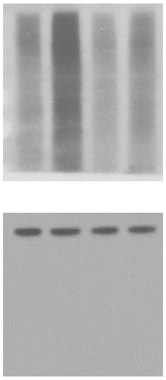

Fig. 3A

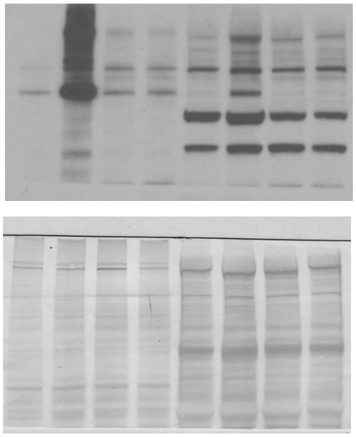

Fig. 3B

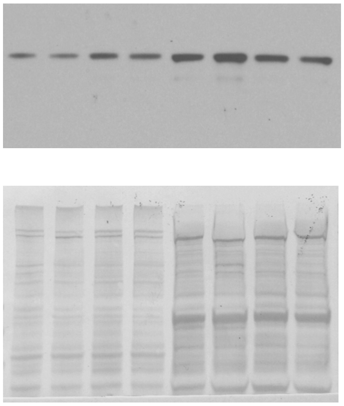

Fig. 3C

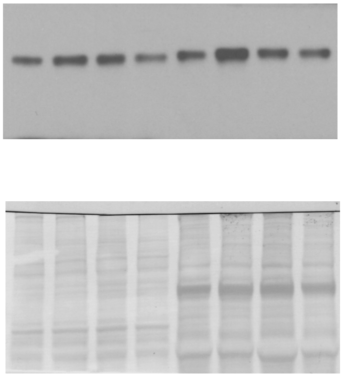

Fig. 3C
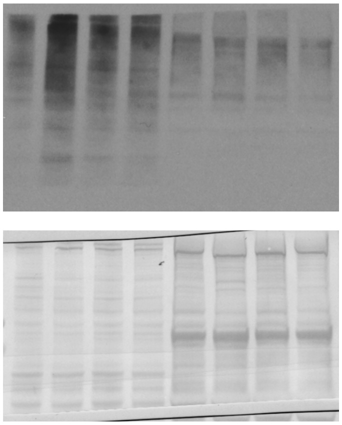

Fig. 4A
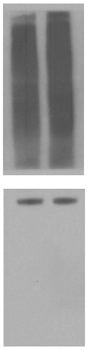

Fig. 4B
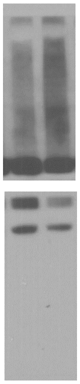

Fig. 4C
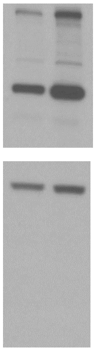

Fig. 4D
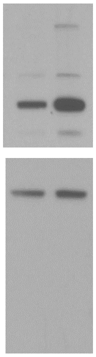

Fig. 4E
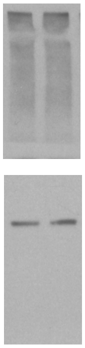

Fig. 4F
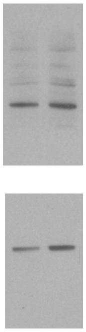

Fig. 5A
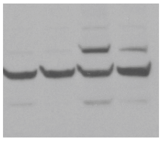

Fig. 5B
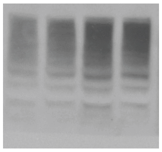

Fig. 5C
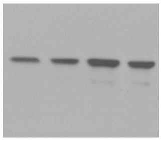

Fig. 5D
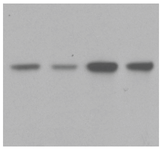

Fig. 8A
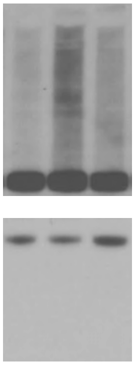

Fig. 8B
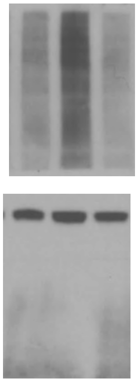

Fig. 8C
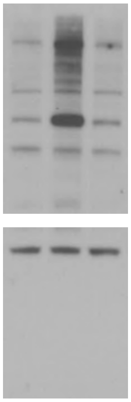

Fig. 8D
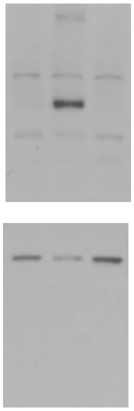

Fig. 8E

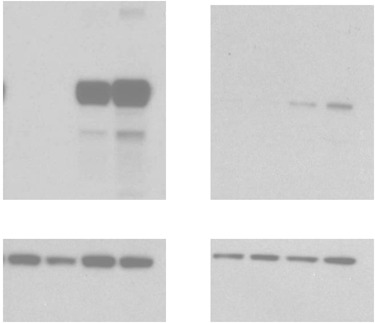

Fig. 8F

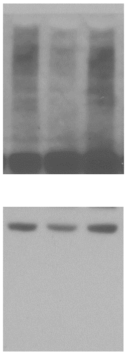

Fig. 8G

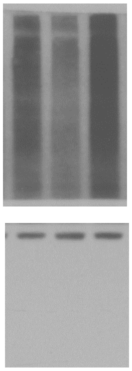

Fig. 9A

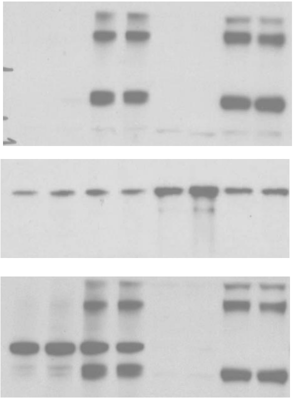

Fig. 9C

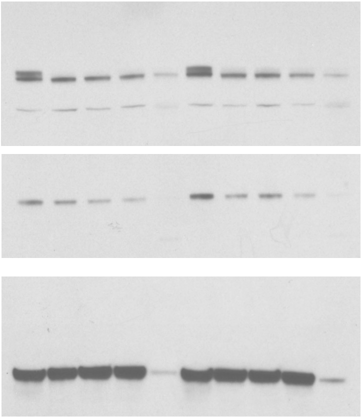

Supplement: S4 Data — (PDF) [file pgen.1008859.s014.pdf]
